# Preclinical Development of a Genetically Engineered Albumin‐Binding Nanoparticle of Paclitaxel

**DOI:** 10.1002/smsc.202400153

**Published:** 2024-09-25

**Authors:** Soumen Saha, Samagya Banskota, Parisa Yousefpour, Jeffrey L. Schaal, Nikita Zakharov, Jianqiao Liu, Michael Dzuricky, Ziwei He, Stefan Roberts, Xinghai Li, Ashutosh Chilkoti

**Affiliations:** ^1^ Department of Biomedical Engineering Pratt School of Engineering Duke University Durham NC 27708 USA

**Keywords:** albumin‐binding domains, cancer, elastin‐like polypeptides, nab‐paclitaxel, preclinical drug development, recombinant nanoparticles

## Abstract

Nab‐paclitaxel (Abraxane), an albumin‐bound solvent‐free paclitaxel (PTX) formulation that takes advantage of the endogenous albumin transport pathway, is the current gold standard for treatment of solid tumors with PTX. However, nab‐paclitaxel has several limitations, including complex manufacturing, immunogenicity, slow drug‐release, and a narrow therapeutic window. Nevertheless, no other PTX formulation has gained the Food and Drug Administration approval since Abraxane's 18‐year reign. Addressing these concerns, herein, a PTX‐loaded nanoparticle of a recombinant polypeptide that—like nab‐paclitaxel—capitalizes on the long in vivo half‐life of albumin is reported. This genetically engineered nanoparticle packages PTX in the core of the nanoparticle and displays an albumin‐binding domain on the exterior of the nanoparticle. Upon in vivo administration, the drug‐loaded nanoparticle binds albumin with nanomolar affinity, and acquires an albumin‐corona, which eliminates the need to use exogenous albumin. The nanoparticles can be stored at subzero temperature as lyophilized powder without any cryoprotectants for upto a year and can be reconstituted on‐demand in aqueous buffer at high concentration, thus greatly simplifying formulation processes. These albumin‐binding nanoparticles improve the therapeutic window by at least twofold compared to nonalbumin‐binding counterpart and outperform nab‐paclitaxel in multiple murine tumor models, results that have been independently replicated by a contract research organization.

## Graphical abstract

Genetically engineered paclitaxel nanoparticles bind endogenous serum albumin post administration, leveraging albumin's long half‐life to improve circulation time, drug exposure, and therapeutic window. These albumin‐binding nanoparticles double the therapeutic window compared to nonalbumin‐binding counterparts and outperform nab‐paclitaxel in various murine tumor models, with results independently confirmed by a contract research organization.

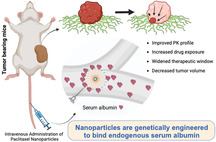

## 1 Introduction

A major challenge in the clinical development of delivery systems for chemotherapeutics—and the many other classes of cancer drugs—is the lack of robustness of the data,^[^ ^1^, ^2^ ^]^ which is rooted in several causes, the most important of which in our opinion is the lack of robust preclinical models that recapitulate human disease and the lack of consistent and universal guidelines on how such preclinical models should be used and evaluated. This has led to many studies that have shown promising preclinical efficacy in a single—usually academic laboratory—but have not been replicated by disinterested third parties elsewhere. While the development of better animal and in vitro models is the subject of vigorous debate and research (and one that we do not address in this study), the lack of replicability between laboratories for the same drug and formulation is a serious concern for the field and one that needs to be urgently addressed. The stability of the formulation is also important for the deployment of new delivery formulations for small molecule chemotherapeutics, as formulations that need a cold chain for transport or require storage at low temperatures also greatly limit the utility of these new formulations.

This article addresses these two major challenges in the preclinical development of new formulations of chemotherapeutics—their lack of replicability across laboratories and their poor stability—in the context of a new delivery system that we have developed for the delivery of a chemotherapeutic—paclitaxel (PTX)—for frontline treatment of diverse solid tumors. Because the solvent used in the commercial formulation of PTX—a 1:1 solution of Kolliphor EL, formerly known as Cremophor EL and dehydrated ethanol—result in hypersensitivity reactions in patients,^[^ ^3^, ^4^, ^5^ ^]^ it necessitates pretreatment with corticosteroids and antihistamines.^[^ ^6^ ^]^ Kolliphor EL can also limit clinical efficacy,^[^ ^7^ ^]^ and intratumoral accumulation of PTX.^[^ ^8^ ^]^ Hence, nab‐paclitaxel (Abraxane),^[^ ^8^ ^]^ an albumin‐bound solvent‐free PTX nanoparticle formulation that takes advantage of the endogenous albumin transport pathway serves as the current gold standard for treatment of solid tumors with PTX. However, nab‐paclitaxel also suffers from several limitations, including complex manufacturing steps,^[^ ^9^ ^]^ potential immunogenicity,^[^ ^9^ ^]^ strict regulations regarding the use of exogenous albumin, a slow and passive drug‐release profile resulting from the physical encapsulation of PTX within nanoparticles,^[^ ^10^ ^]^ and a narrow therapeutic window.^[^ ^11^, ^12^, ^13^, ^14^ ^]^

In previous studies, we developed a synthetic intrinsically disordered protein, a chimeric polypeptide (CP) for the delivery of chemotherapeutics as a nanoparticle. Originally demonstrated with the delivery of Doxorubicin,^[^ ^15^ ^]^ we adapted this design to deliver PTX.^[^ ^16^ ^]^ We showed that it was superior to the Cremophor formulation of PTX and nab‐paclitaxel. However, CP‐PTX was only superior to nab‐paclitaxel at a high dose that was close to its maximum tolerated dose (MTD). It also suffered from poor colloidal stability, as prolonged storage at −20 °C led to aggregation of the nanoparticles. We have also shown that a CP‐doxorubicin nanoparticle that present an albumin‐binding protein domain shows improved pharmacokinetics, tumor accumulation, and efficacy compared to nanoparticles that do not bind albumin.^[^ ^17^ ^]^

**Scheme 1:**
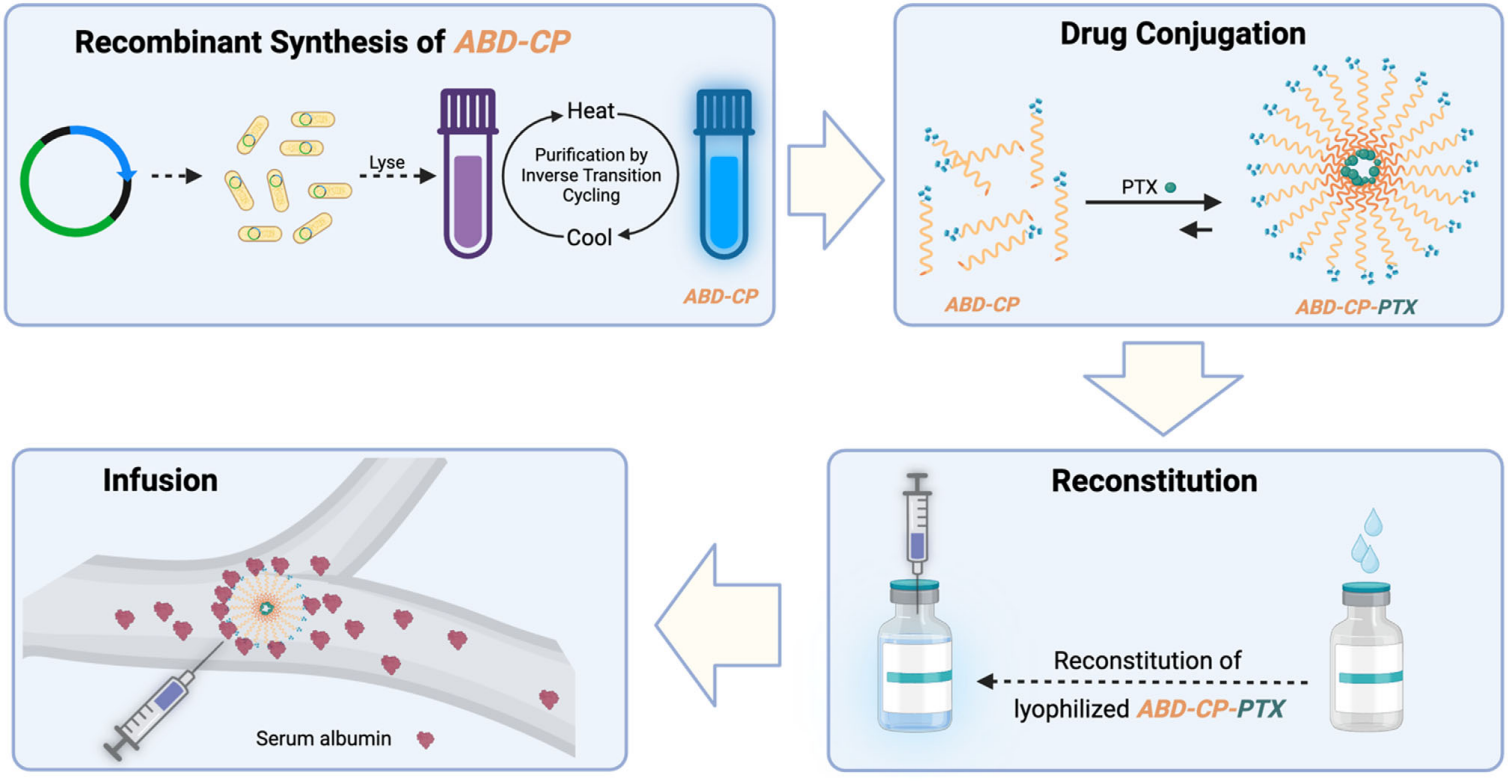
Schematic representation of the synthesis and deployment of a genetically engineered albumin‐binding nanoparticle with a PTX cargo. An albumin‐binding domain (ABD) is fused to a CP at the gene level and is purified from *E. coli* at the gram scale or greater using a nonchromatographic method known as inverse transition cycling. Upon conjugation of ≈2 copies of PTX per CP chain, ABD‐CP‐PTX self‐assembles into monodisperse nanoparticles due to the hydrophobicity of the conjugated PTX. The conjugate can be lyophilized and stored at subzero temperatures without any cryoprotectants or additives for at least a year. The lyophilized nanoparticles can be reconstituted in physiological saline for in vivo administration. Because the exterior of the nanoparticles is decorated with an albumin‐binding domain, ABD‐CP‐PTX nanoparticles bind endogenous albumin with high affinity upon systemic administration, which creates an albumin corona on the nanoparticle, and thus prolongs the half‐life of the drug.

Building on these previous studies and motivated by the fact that despite the limitations of nab‐paclitaxel no other formulation has gained the Food and Drug Administration (FDA) approval in the last two decades since nab‐paclitaxel was approved, we report herein an albumin‐binding CP‐PTX nanoparticle (**Scheme** **1**) that can be formulated and stored for an extended duration of time as a lyophilized powder and which, upon resuspension, has excellent colloidal stability over a wide temperature range. In addition, even at a moderate dose of 25 mg kg^−1^ B.W. of PTX eqv. CP‐PTX needs a slow infusion because of its high viscosity, whereas ABD‐CP‐PTX can be administered as a bolus injection because of its lower viscosity. This formulation also has a therapeutic window that is at least twofold greater than the parental CP‐PTX formulation or nab‐paclitaxel and outperforms nab‐paclitaxel in multiple murine tumor models, results that have been independently validated by a contract research organization, which augurs well for its translation to the clinic.

## 2 Results

### 2.1 Synthesis and Characterization of Albumin Binding Polypeptide

The recombinantly engineered CP that acts as a building block of the PTX‐loaded nanoparticle consists of three genetically encoded polypeptide blocks. The first block of the CP is an elastin‐like polypeptide (ELP),^[^ ^18^, ^19^, ^20^ ^]^ a genetically encoded synthetic intrinsically disordered protein that has excellent biocompatibility and is not immunogenic.^[^ ^20^ ^]^ To ensure that the drug conjugate has sufficient amphiphilicity to self‐assemble into nanoparticles —micelles—upon drug conjugation, we chose a hydrophilic ELP that consists of 160 repeats of the Val‐Pro‐Gly‐Xaa‐Gly, where Xaa is Val:Gly:Ala in a 7:8:1 ratio. This length of the ELP also ensures that the MW of the final conjugate remains close to the glomerular filtration threshold of 60 kDa.^[^ ^21^ ^]^ The second block—a drug attachment domain—is a short cysteine‐rich (Gly‐Gly‐Cys)_8_ peptide segment at the C‐terminus of the ELP that provides multiple thiol moieties for drug attachment. We have shown that attachment of multiple copies of hydrophobic small molecules to the Cys residues in the drug attachment domain imparts sufficient amphiphilicity to trigger self‐assembly of the CP‐drug conjugate into spherical micelles.^[^ ^15^, ^22^ ^]^

The third block is an ABD that is fused at the gene level to the CP at the opposite end of the drug attachment domain. The fusion of an ABD to a CP is termed ABD‐CP. The ABD used in this study is an engineered variant of a native ABD derived from streptococcal protein G21, and is a forty six amino acid long, three‐helix bundle protein domain that has several orders of magnitude higher affinity for human serum albumin than the native version.^[^ ^23^, ^24^, ^25^ ^]^ The amino acid sequence of the ABD‐CP and CP is provided in Table S1, Supporting Information. We chose an ABD instead of other ligands that bind albumin like fatty acids,^[^ ^26^ ^]^ Evan's blue,^[^ ^27^ ^]^ or oligonucleotides,^[^ ^28^ ^]^ to create albumin‐coated nanoparticles, because ABDs are genetically encodable and express at a high level in *E. coli* and have high aqueous solubility, as do ELPs, which allows the entire drug carrier to be synthesized recombinantly in *E. coli*.^[^ ^25^ ^]^ ABDs are also structurally robust and stable,^[^ ^29^ ^]^ as seen by their high melting temperature and their ability to withstand denaturation by extremes of pH.^[^ ^30^ ^]^ These features suggest that ABDs can preserve their conformation and activity under the processing conditions required to purify the peptide‐drug conjugate, such as high temperature (55 °C) and the addition of organic cosolvents.^[^ ^17^, ^31^ ^]^ We also synthesized a negative control for albumin binding—a CP that is not fused to an ABD at its N‐terminus.

**Figure 1:**
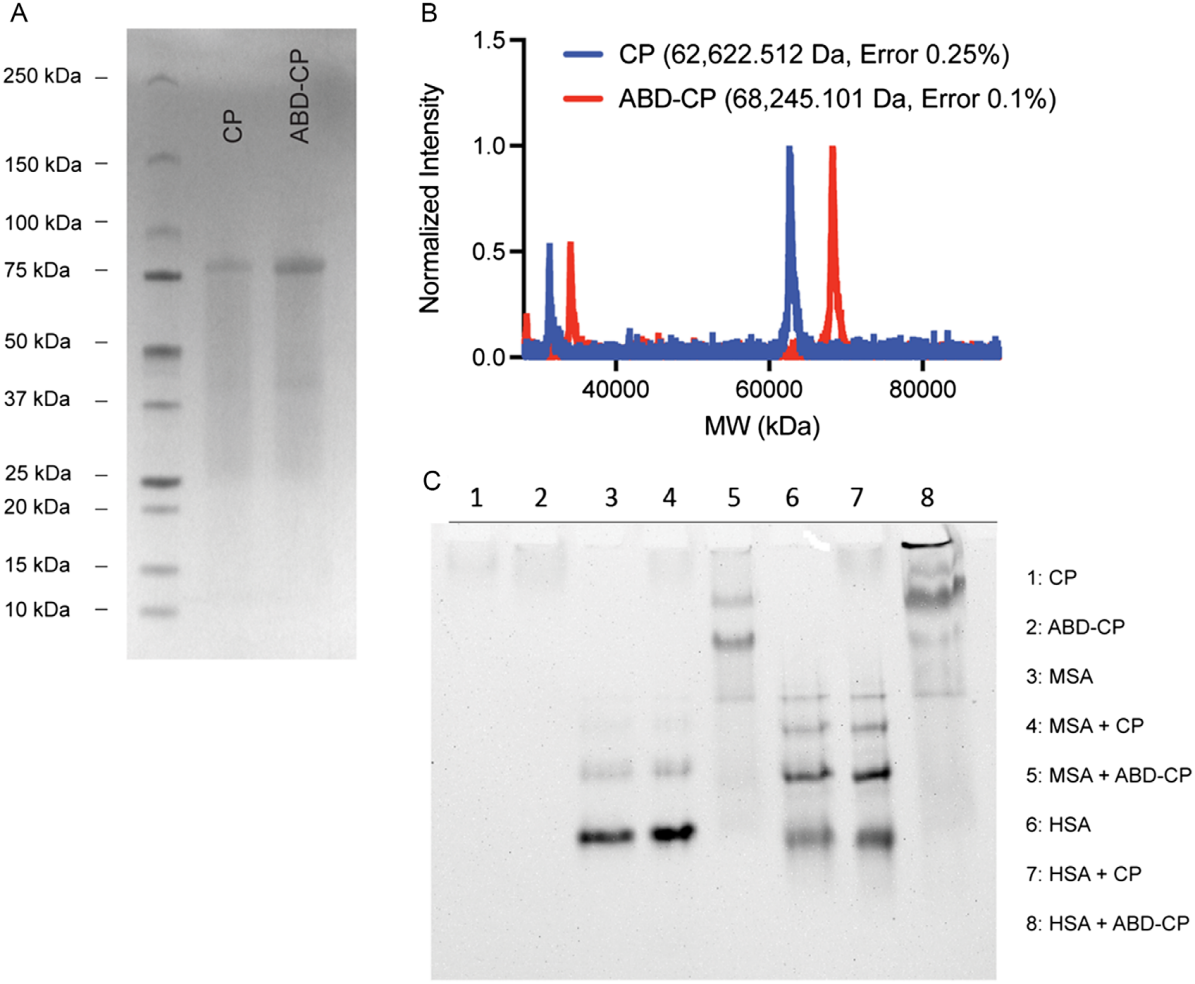
Characterization of the recombinant albumin‐binding CP. A) SDS‐PAGE of ABD‐CP and CP shows the high purity of both polypeptides. B) MALDI‐TOF MS spectra of ABD‐CP and CP analysis show that the molecular weight (MW) of both polypeptides matches their theoretical value. C) Native‐PAGE of ABD‐CP‐PTX in the presence of serum albumin (lane 5 and 8) indicates that it binds MSA and HSA.

Using a nonchromatographic technique, known as inverse transition cycling,^[^ ^32^ ^]^ the recombinant ABD‐CP and CP were purified from *E. coli* with >100 mg mL^−1^ yield from shaker flask culture and with >95% purity, as assessed by sodium dodecyl sulfate‐polyacrylamide gel electrophoresis (SDS‐PAGE) (**Figure** **1**A). The molecular weight of the ABD‐CP, as measured by matrix‐assisted laser desorption/ionization time‐of‐flight mass spectrometry (MALDI‐TOF‐MS), closely matched its theoretical value, as did that of the CP (Figure 1B). For preliminary assessment of the albumin‐binding capability of the ABD‐CP, native PAGE was used. ABD‐CP and CP do not migrate on native‐PAGE whereas human serum albumin (HSA) and mouse serum albumin (MSA) migrate into the gel toward the cathode due to their high negative charge (Figure 1C). However, when mixed, the binding of HSA or MSA to the ABD‐CP increases the overall size and decreases the charge to molecular weight ratio, thus impeding the electrophoretic mobility of the complex on the gel. As the interaction of albumin with ABD‐CP is noncovalent and reversible, the free albumin in equilibrium with the bound complex migrates in the gel, resulting in a smear. In contrast, mixing of the negative control—the CP—with HSA or MSA did not affect the migration pattern of HSA or MSA in native PAGE.

### 2.2 Synthesis and Characterization of PTX‐Containing Nanoparticles

**Figure 2:**
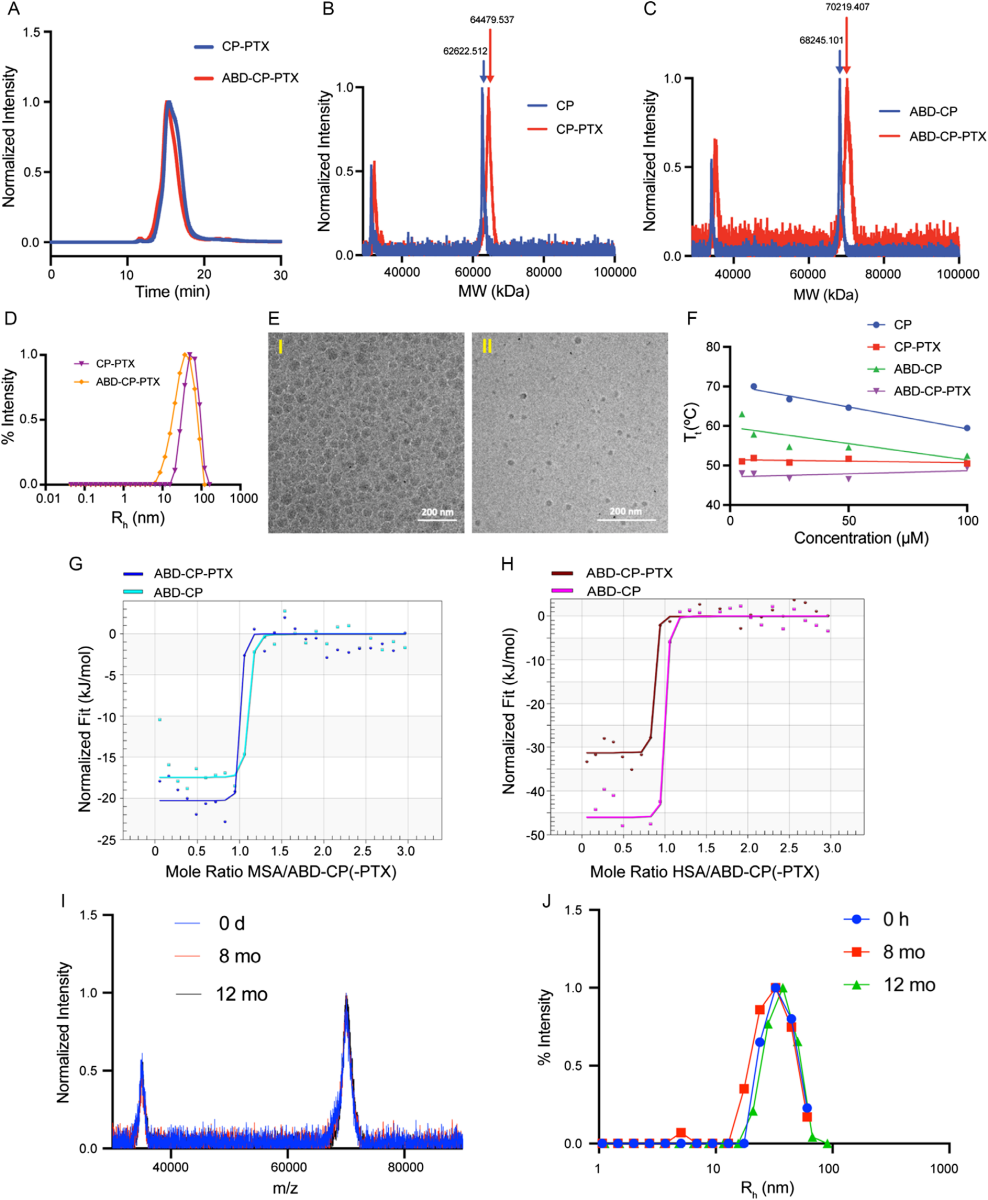
Characterization of the PTX‐loaded nanoparticles. A) Purity of the conjugates as determined by SEC‐HPLC. B,C) MALDI‐TOF‐MS of the PTX conjugates. Drug attachment ratio was determined from the MW differences between conjugate and the native polypeptide. D) Hydrodynamic radius (*R*_h_) of the conjugates in PBS suggests formation of nanoparticles, which is corroborated by Cryo‐TEM E)—I) CP‐PTX and II) ABD‐CP‐PTX. F) Temperature‐programmed turbidimetry of the CP and ABD‐CP and their PTX conjugates in PBS as a function of polypeptide concentration. The concentration‐independent *T*_t_ of the PTX conjugates suggests that they are nanoparticles at all concentrations. Isothermal calorimetric titration of ABD‐CP‐PTX nanoparticles with MSA G) and HSA H) in PBS at 37 °C. The solid line indicates the best‐fit binding of the binding isotherm. The integrated heat data were fit to a single‐site binding model and the binding stoichiometry (*N*) and dissociation constant (*K*_D_) were calculated, as shown in Table 2. I,J) Long‐term stability of ABD‐CP‐PTX. ABD‐CP‐PTX can be lyophilized and stored without any cryoprotectants and/or excipients for at least a year without any loss of API as evident from the MALDI‐TOF analysis (I) and can be reconstituted in aqueous buffer as monodisperse nanoparticles after a year of storage at –80 °C, as seen by DLS (J).

The C‐terminus of the ABD‐CP and CP containing the drug attachment domain—(CGG)_8_—was used to covalently conjugate the 2′‐OH of PTX via an acid labile hydrazone linker, as described previously^[^ ^33^ ^]^ (Figure S1, Supporting Information). Briefly, the 2′‐OH was first coupled with levulinic acid (LEV) to introduce a ketone functionality. The ketone‐containing product was reacted with n‐maleimidocaproic acid hydrazide (EMCH) to incorporate the pH‐sensitive hydrazone linker while simultaneously activating the PTX with a maleimide group. The ESI‐MS and proton NMR spectrogram are provided in the supplementary (Figure S2, Supporting Information), and closely match the previous data.^[^ ^16^ ^]^ The maleimide‐activated PTX was then conjugated to the cysteine residues of ABD‐CP or CP by a Michael addition reaction. The conjugate was purified at the milligram scale by centrifugal ultrafiltration using Amicon spin filters and at the gram scale by tangential flow filtration. Size exclusion chromatography (SEC) on a high‐pressure liquid chromatography (HPLC) system of ABD‐CP‐PTX and CP‐PTX showed that both conjugates were >95% pure (**Figure** **2**A). MALDI‐TOF‐MS showed that both conjugates had ≈2 PTX molecules per polypeptide, as determined by the mass difference between the conjugate and the parent polypeptide (Figure 2B,C). Characterization data of the ABD‐CP‐PTX and CP‐PTX are summarized in **Table** **1**.

**Table 1:** Summary of characterization for recombinant PTX‐nanoparticles.

Conjugates	Puritya) [%]	Drug loadingb) [%wt/wt]	DLS Analysisc)		SLS Analysisc)			CACd) [μm]	*T*_t_ [°C]e)
			*R*_H_ [nm]	% Pd	*R*_G_ [nm]	MW_Agg_ [mda]	*N*_agg_		
ABD‐CP‐PTX	>95	≈2 (3.4%)	44.1 ± 4.7	27%	42.83	4.55	64.5	0.92	≈47
CP‐PTX	>95	≈2 (3.7%)	52.4 ± 2.9	31%	38.35	1.06	16.2	1.3	≈51

a) Purity was assessed with SEC‐HPLC;

b) Drug loading was assessed from MALDI‐TOF and expressed as number of PTX molecules per polypeptide chain;

c) Light scattering experiments were performed at 37 °C in PBS;

d) Determined using pyrene as a hydrophobic fluorescent probe;

e) Transition temperature was measured by thermal turbidimetry at 5–100 μm in PBS.

Dynamic light scattering (DLS) of both conjugates revealed that conjugation of PTX—a highly hydrophobic molecule with a LogP of 4.7—to CP and ABD‐CP triggered spontaneous self‐assembly of the conjugates into nanoparticles. ABD‐CP‐PTX and CP‐PTX formed near‐monodisperse nanoparticles with an average hydrodynamic radius (*R*_h_) of ≈44 and ≈52 nm, respectively (Figure 2D). Interestingly, analysis of the partial Zimm plot obtained from static light scattering (SLS) revealed that the radius of gyration (*R*_g_) was ≈42 nm for ABD‐CP‐PTX and ≈38 nm for CP‐PTX. The *R*_g_/*R*_h_ ratios of the nanoparticles are consistent with spherical nanoparticles.^[^ ^16^ ^]^ The aggregation number (*N*_agg_) of ABD‐CP‐PTX is 64.5 and that of CP‐PTX is 16.2 (Figure S3, Supporting Information). The fourfold higher aggregation number of ABD‐CP‐PTX relative to CP‐PTX indicates that ABD‐CP‐PTX micelles are more densely packed than the CP‐PTX micelles.

These results were corroborated by cryo‐TEM that shows that the ABD‐CP‐PTX nanoparticles are much smaller nanoparticles (18.2 ± 3.6 nm, *n* = 30) compared to CP‐PTX (45 ± 8.5 nm, *n* = 35) and that they both have a spherical morphology (Figure 2E). It is important to note that due to the low electron density of the highly hydrated polypeptide corona and poor scattering contrast of the constituent elements with a low atomic number, cryo‐TEM only allows visualization of the hydrophobic core with a low level of contrast. The relatively higher contrast in the cryo‐TEM images of the ABD‐CP‐PTX nanoparticles is also suggestive of their denser packing than the CP‐PTX nanoparticles. In contrast, DLS measures the size‐dependent scattering behavior of the nanoparticle that correlates with the MW of the highly hydrated polypeptide corona, which is very similar for CP‐PTX and ABD‐CP‐PTX.

The critical aggregation concentration (CAC) of the drug‐modified nanoparticles was measured by fluorescence spectroscopy using pyrene as a probe. As the concentration of the conjugate decreases, the fluorescence intensity ratio of the 370–373 nm peak to the 381–384 nm peak (I1/I3) increases, reflecting nanoparticle disassembly and release of pyrene from the hydrophobic core of the nanoparticles into the aqueous environment (Figure S4, Supporting Information). The CAC, defined as the concentration at the inflection point in the I1/I3 fluorescence emission ratio, is 0.92 μm for ABD‐CP‐PTX while that of CP‐PTX is 1.3 μm.

Next, we determined the transition temperature (*T*_t_) of the drug conjugates and parent polypeptides by temperature‐programmed turbidimetry, as the CPs contain an ELP that exhibits lower critical solution temperature transition phase behavior.^[^ ^18^ ^]^ As shown in Figure 2F and in Table 1, both polypeptides and the drug conjugates phase separate at a temperature that is higher than body temperature (37 °C). An important observation is that although the *T*_t_ of the parent polypeptides is inversely proportional to their concentration, the *T*_t_ of their PTX conjugates is independent of their concentration, which is a signature of nanoparticle formation.^[^ ^16^ ^]^ Finally, the binding of albumin by ABD‐CP‐PTX and CP‐PTX was confirmed by isothermal titration calorimetry. Analysis of the calorimetric data revealed that ABD‐CP‐PTX nanoparticles bind to both MSA (Figure 2G) and HSA (Figure 2H) with near equimolar stoichiometry and with a nanomolar affinity. The experimentally determined dissociation constant (*K*_D_) of ABD‐CP‐PTX nanoparticles was 4.4 nm for MSA and 5.6 nm for HSA (**Table** **2**). In contrast, CP‐PTX does not show strong binding to MSA or HSA (Figure S5, Supporting Information). Collectively, these data suggest that both CP‐PTX and ABD‐CP‐PTX can self‐assemble into monodisperse spherical nanoparticles in aqueous buffer and that only ABD‐CP‐PTX binds to albumin with nanomolar affinity.

**Table 2:** Calculated thermodynamic parameters of ABD‐CP‐PTX from isothermal titration calorimetry.

Conjugates‐albumin interaction	MSA		HSA	
Thermodynamic parametersa)	ABD‐CP	ABD‐CP‐PTX	ABD‐CP	ABD‐CP‐PTX
Binding stoichiometry (*N*)	1.05	0.95	0.95	0.82
Dissociation Constant (*K*_D_) [nm)	16.3	4.37	6.47	5.58

a) The binding and thermodynamic parameters—binding constant (KD) and number of binding sites (N)—were computed by non‐linear curve fitting of the data to a single site binding model using the Origin Lab software provided with the VP‐ITC calorimeter.

### 2.3 Stability

Long‐term storage of a drug product in a lyophilized form is important due to ease of handling and transport of the lyophilized drug. Indeed, substantial research is undertaken during drug development to optimize the lyophilization process of a drug formulation by the use of cryoprotectants, optimization of the buffer, lyophilization temperature, pressure, ramp rate, and the method of reconstitution.^[^ ^34^, ^35^ ^]^ Because the ABD and CP can tolerate a harsh chemical environment and the hydrazone bond is only labile at low pH, we hypothesized that the ABD‐CP conjugate can be lyophilized and stored without any cryopreservatives or additives. We systematically assessed the stability of the lyophilized conjugates at room temperature (RT) and −20 °C by monitoring drug release by SEC‐HPLC, and size by DLS. As seen in Figure S6, Supporting Information, both drug formulations released less than 5% PTX after 3 months at both RT and −20 °C, indicating that the hydrazone bond can withstand lyophilization, freeze‐thaw, and reconstitution. However, the size profile of reconstituted lyophilized CP‐PTX in PBS resulted in a highly polydisperse population of nanoparticles in solution (Figure S6, Supporting Information). A regularization fit of the DLS autocorrelation curves showed the appearance of >100 nm radius particles after 14 days of storage. ABD‐CP‐PTX, in contrast, can be resolubilized as a monodisperse nanoparticle even after 3 months of storage with no aggregation into larger particles (Figure S6, Supporting Information) and was stable for at least one year at −80 °C (Figure 2I,J). MALDI‐TOF‐MS of ABD‐CP‐PTX at different timepoints do not show a significant difference and superimpose upon each other (Figure 2I). Analysis of the mass suggests less than 1% loss of attached PTX from ABD‐CP‐PTX even after 370 days of storage at −80 °C. ABD‐CP‐PTX can also be resolubilized as monodisperse nanoparticles (Figure 2J) with no aggregation. Moreover, DLS analysis of a one‐year‐old sample in the presence of HSA at physiological temperature and pH revealed that the size of the ABD‐CP‐PTX nanoparticles increases significantly in the presence of albumin due to efficient binding of HSA, whereas the size remains virtually the same for the CP‐PTX nanoparticle (Figure S7, Supporting Information). A regularization fit of the scattering data indicates presence of two populations of CP‐PTX. The smaller particle (≈4.1 nm) is the unbound albumin, which is consistent with the size of free albumin, and indicates the lack of affinity of CP‐PTX nanoparticles for albumin. In contrast, the ABD‐CP‐PTX nanoparticles incubated with albumin show only one population, which is 64% larger (≈57 nm) than the native nanoparticle. Moreover, this increase in size remains the same after 4 h of incubation indicating the strong affinity of ABD‐CP‐PTX toward albumin and the stability of the nanoparticles in the presence of albumin.

In addition, we analyzed the short‐term solution phase stability of ABD‐CP‐PTX after reconstitution in aqueous buffer. This is important to demonstrate the integrity of the nanoparticle during onsite administration and handling. As shown in Figure S8, Supporting Information, resolubilized ABD‐CP‐PTX remains a stable nanoparticle at RT for at least 24 h. Collectively, these results establish that the ABD‐CP‐PTX formulation can be lyophilized and stored at various temperatures without any cryoprotectants or excipients and can be reconstituted in aqueous buffer on‐demand as nanoparticles, without any significant loss of the active pharmaceutical ingredient—PTX.

### 2.4 Drug Release and Cytotoxicity

**Figure 3:**
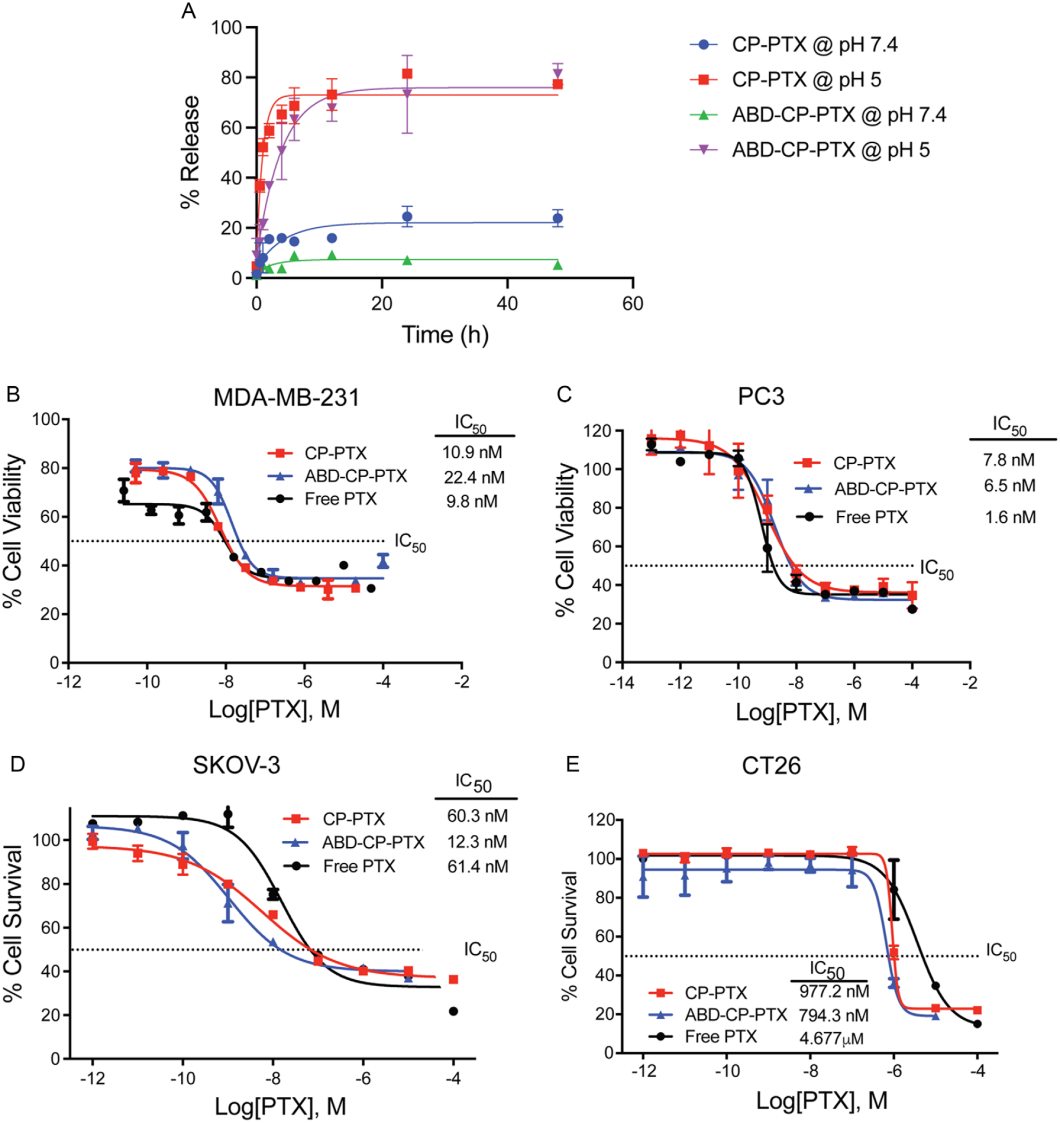
In vitro potency of the ABD‐CP‐PTX nanoparticles. A) Drug release profile of PTX nanoparticles (mean ± SD). B) Cell viability for ABD‐CP‐PTX, CP‐PTX, and free PTX after 72 h of treatment of MDA‐MB‐231, C) PC3, D) SKOV‐3, and E) CT26 cell lines (mean ± SEM, *n* = 3).

To measure their pH‐responsive drug release, ABD‐CP‐PTX and CP‐PTX nanoparticles were incubated with PBS (pH 7.4) and acetate buffer (pH 5.3) at 37 °C. At a specified timepoint, released drug—due to acid‐promoted cleavage of the hydrazone moiety—was separated from the conjugate by SEC‐HPLC and quantified by the absorbance at 228 nm (absorbance maximum of PTX) (**Figure** **3**A). Both CP‐PTX and ABD‐CP‐PTX released more than 75% of the drug within the first 12 h at pH 5.3. Interestingly, at pH 7.4 release of PTX from the ABD‐CP‐PTX nanoparticles is significantly slower than that of CP‐PTX. At 24 h, the percentage of PTX released from ABD‐CP‐PTX is ≈3.5‐fold less than that of CP‐PTX (*P* < 0.001, Sidak's test) indicating that PTX is better protected within ABD‐CP‐PTX nanoparticles at physiological pH. This may be related to the higher packing density of ABD‐CP‐PTX (*N*_agg_ ≈ 65) compared to CP‐PTX (*N*_agg_ ≈ 16). An equally important observation is that within this time frame (48 h) nab‐paclitaxel only releases ≈25% of its PTX cargo and the release of 50% of PTX from nab‐paclitaxel may take up to a week.^[^ ^36^ ^]^

Next, we chose four widely different cancer cell lines to evaluate the cytotoxicity of the PTX conjugates. PTX was used as free drug control in this study. We chose MDA‐MB‐231, a human triple‐negative breast cancer cell line (TNBC) as the first cell line for treatment with the ABD‐CP–PTX nanoparticles, because PTX is used to treat patients with TNBC. A total of 10–15% of breast cancers are triple negative and are characterized by a lack of expression of the estrogen receptor, progesterone receptor, and the HER‐2 gene.^[^ ^37^ ^]^ TNBC presents a difficult clinical challenge, as it afflicts younger patients, is refractory to chemotherapy, and displays a shorter median time to relapse and death than other subtypes of breast cancer.^[^ ^37^ ^]^ We also compared the anti‐cancer efficacy of the PTX conjugates against the PC3 cell line. PC3 was chosen because it is a model for highly metastatic, androgen‐independent prostate cancer.^[^ ^38^ ^]^ Androgen receptor‐negative prostate tumors do not respond to traditional hormone therapy, and therefore, taxanes are used as the first line of treatment.^[^ ^39^ ^]^ Hence, the treatment of mice bearing MDA‐MB‐231 and PC3 tumors with ABD‐CP‐PTX nanoparticles provides a stringen*t* test of its utility in assessing the in vitro efficacy of the drug.

After 72 h of treatment, the proliferation of MDA‐MB‐231 and PC3 cells was significantly inhibited for the free drug and conjugates (Figure 3B,C). The half‐maximal inhibitory concentration (IC_50_)—defined as the concentration of PTX equivalent required for 50% inhibition of cells—was 10.9 nm and 7.8 nm for CP‐PTX, 22.4 nm and 6.5 nm for ABD‐CP‐PTX, and 9.8 and 1.6 nm for free PTX against MDA‐MB‐231 and PC3 cells, respectively. We also measured the IC_50_ value of the nanoparticles against SKOV3, a human ovarian cancer cell line and CT26, a mouse colon cancer cell line. ABD‐CP‐PTX nanoparticles display a ≈5‐fold lower IC_50_ than the free drug in both cell lines (Figure 3D,E). These data are notable for several reasons. First, they show that ABD‐CP‐PTX remains effective against a broad spectrum of neoplastic cell lines. Second, covalent conjugation of small molecule drugs to polymers frequently results in a significant decrease in their in vitro potency^[^ ^17^ ^]^; in contrast, conjugation PTX to the CP or ABD‐CP via an acid‐labile bond does not decrease the in vitro potency of the drug.

### 2.5 In Vivo Tumor Regression Efficacy at Maximum Feasible Dose

**Figure 4:**
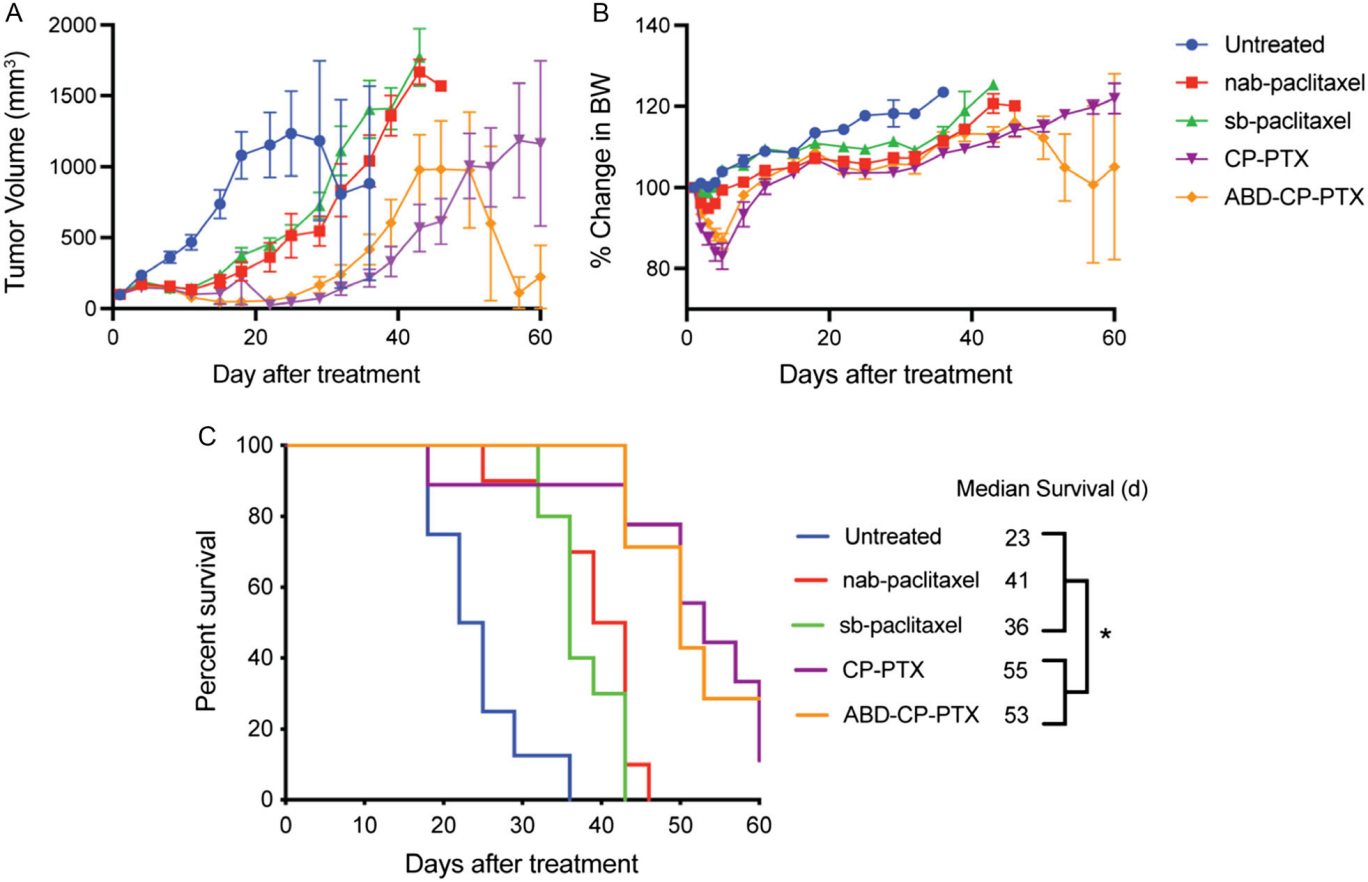
In vivo tumor regression of ABD‐CP‐PTX nanoparticles in MDA‐MB‐231 orthotopic model at maximum feasible dose. MDA‐MB‐231 tumor cells were implanted orthotopically and allowed to grow to ≈100 mm^3^. Mice were treated on day 0 with a single dose of PTX (sb‐paclitaxel) at 25 mg Kg^−1^ B.W PTX equivalent, and CP‐PTX, ABD‐CP‐PTX, and nab‐paclitaxel at 50 mg Kg^−1^ B.W PTX equivalent. A) Tumor volume up to day 60. B) Change in percentage body weight after treatment. C) Cumulative survival of mice up to day 60. Data represented as mean ± SEM. *P < 0.05.

Another big issue in the translation of new drugs and delivery systems to the clinic is the lack of reproducibility of the data across laboratories. A recent meta‐analysis suggests that more than 50% of academic cancer research is irreproducible.^[^ ^1^, ^2^ ^]^ To directly address this issue, we outsourced the first set of tumor regression experiments to a contract research organization—Charles River laboratories. In this experiment, mice with orthotopic MDA‐MB‐231 tumors were intravenously treated with a single dose of ABD‐CP‐PTX nanoparticles, CP‐PTX nanoparticles, or nab‐paclitaxel at a maximum feasible dose of 50 mg Kg^−1^ B.W PTX equivalent, or Kolliphor‐ethanol solvent based PTX (sb‐paclitaxel) at its MTD of 25 mg Kg^−1^ B.W PTX equivalent.^[^ ^16^ ^]^ The maximum feasible dose of 50 mg Kg^−1^ B.W PTX equivalent for the three nanoparticles was dictated by the maximum solubility of the CP‐PTX and ABD‐CP‐PTX conjugates within an acceptable dosing volume. On day 18 after treatment, ABD‐CP‐PTX‐treated mice had a mean tumor volume of 119 mm^3^ (*n* = 10) versus 261 mm^3^ (*n* = 10) for nab‐paclitaxel, 212 mm^3^ (*n* = 9) for CP‐PTX, 375 mm^3^ (*n* = 10) for sb‐paclitaxel (Tukey's test; *P* < 0.05) and 1081 mm^3^ (*n* = 9) for untreated controls (Tukey's test; *P* < 0.0001) (**Figure** **4**A). The % body weight loss remained well below the threshold 20% cut‐off that indicates significant systemic toxicity for all treatments (Figure 4B). Interestingly, both CP‐PTX and ABD‐CP‐PTX demonstrate equal efficacy (*P*, ns, Tukey's test) beyond week 3 and both outperformed nab‐paclitaxel and sb‐paclitaxel on any given day (Tukey's test; *P* < 0.05), which correlated with improved animal survival (Figure 4C). The median survival time for untreated mice was 23 days, and treatment with sb‐paclitaxel increased survival to 36 days (Kaplan–Meier, Wilcoxon test, *P* < 0.0001). Treatment with nab‐paclitaxel further increased the survival to 41 days (Kaplan–Meier, Wilcoxon test, *P* < 0.0001). Treatment with ABD‐CP‐PTX and CP‐PTX increased the survival to 53 and 55 days respectively, an increase of ≈2.4‐fold and ≈1.3‐fold compared to untreated mice and nab‐paclitaxel treated mice, respectively.

### 2.6 Dose‐Dependent Pharmacokinetics and Tumor Accumulation

**Figure 5:**
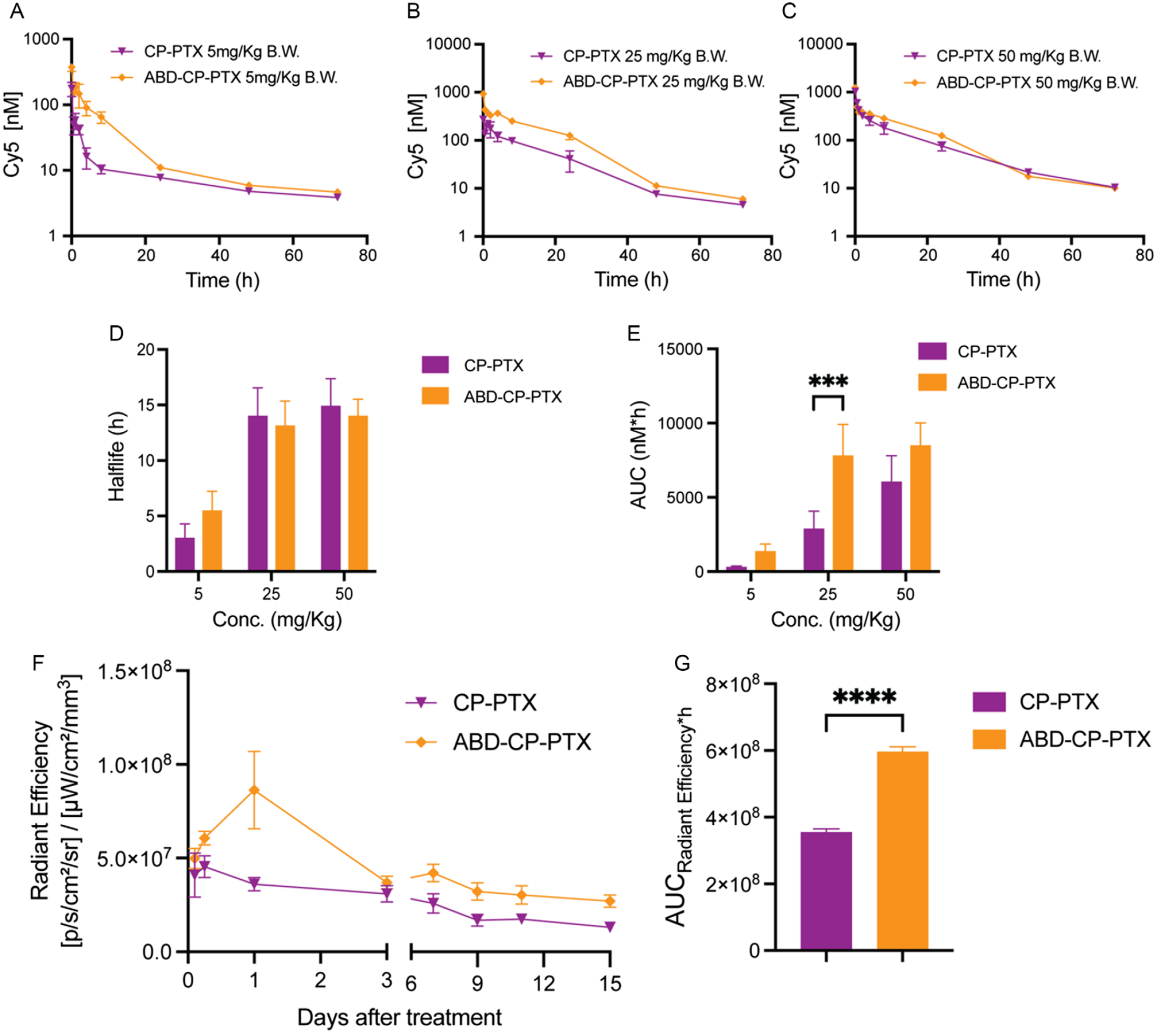
Pharmacokinetics and tumor accumulation of ABD‐CP‐PTX and CP‐PTX nanoparticles. A−C) Plasma concentration of fluorescently labeled nanoparticles in healthy mice treated with a single i.v. injection of ABD‐CP‐PTX and CP‐PTX (5, 25, 50 mg Kg^−1^ B.W. equivalent of PTX). Plasma concentration was fit to a two‐compartment model to determine pharmacokinetic parameters: D) half‐life (*t*_1/2_) and E) area under the curve (AUC) as a function of dose. F,G) Tumor accumulation of ABD‐CP‐PTX and CP‐PTX nanoparticles in MDA‐MB‐231 tumor‐bearing mice intravenously treated with fluorescently labeled PTX nanoparticles at a dose of 25 mg Kg^−1^ B.W. equivalent of PTX. (F) Radiant efficiency as a function of time posttreatment. (G) Total drug exposure of the nanoparticles in the tumor, reported as the AUC of the radiant efficiency versus time data from 2 to 360 h post injection. Data are represented as mean ± SEM (*n* = 3–4). ***P < 0.05 unpaired *t*‐test, ****P < 0.0005 two‐way ANOVA, and Tukey's multiple comparison test.

We next measured the pharmacokinetics of the nanoparticle formulations in mice as a function of PTX dose in‐house at Duke University. The pharmacokinetics were studied in healthy BALB/c mice by intravenously administering cyanine‐labeled PTX‐nanoparticles (Figure S9, Supporting Information) at three different doses. All three doses of PTX‐nanoparticles—5, 25, and 50 mg Kg^−1^ B.W. of PTX equivalent—were formulated so that they had same amount of cyanine dye (50 μm Cy5 analog). The cyanine concentration in blood as a function of time post i.v. injection was quantified by the fluorescence emission of the dye and the data were fit to a two‐compartment model (**Figure** **5**A–C) using PK solver.^[^ ^40^ ^]^ The pharmacokinetic parameters for the different formulations are tabulated in Table S3, Supporting Information. At the lower dose of 5 mg kg^−1^ B.W. equivalent of PTX, ABD‐CP‐PTX nanoparticles displayed a half‐life of 5.5 ± 0.9 h and an AUC of 1458 ± 260 nm h, and these parameters were ≈1.8‐fold and ≈fivefold greater than the half‐life and AUC of CP‐PTX nanoparticles of 3.0 ± 0.7 h and 293 ± 44 nm h, respectively (Figure 5D,E). However, at a higher dose of 25 or 50 mg Kg^−1^ B.W. equivalent of PTX, we found no significant difference between the half‐life of ABD‐CP‐PTX and CP‐PTX nanoparticles (*t*_1/2 _≈ 14 h). The AUC of ABD‐CP‐PTX at 50 mg Kg^−1^ B.W. equivalent of PTX was 8479 ± 772 nm h and is ≈1.4‐fold greater than that of CP‐PTX (6028 ± 888 nm h) that is neither statistically significant (*P* = 0.063) nor therapeutically relevant (Figure 4). Importantly, the AUC of ABD‐CP‐PTX at 25 mg Kg^−1^ B.W. equivalent of PTX was 2.7‐fold higher than that of CP‐PTX (7797 ± 1062 vs 2873 ± 689 nM h), which is statistically significant (*P* = 0.0006).

To test the hypothesis that this statistically significant difference in the AUC at 25 mg Kg^−1^ B.W. equivalent of PTX dose is therapeutically relevant, we next measured the tumor exposure of the cyanine labeled PTX nanoparticles in MDA‐MB‐231 tumor xenograft by noninvasive imaging using an IVIS in vivo imaging system. We calculated the fluorescence flux profile from the treated tumors to evaluate the tumor accumulation over 15 days. ABD‐CP‐PTX treated tumors had a significantly higher fluorescence flux than CP‐PTX tumors at 25 mg Kg^−1^ B.W. equivalent of PTX dose (Figure 5F). The AUC of ABD‐CP‐PTX was ≈twofold higher than the AUC of CP‐PTX nanoparticles (*P* < 0.0001, unpaired *t*‐test), which indicates greater intratumoral accumulation of ABD‐CP‐PTX nanoparticles than CP‐PTX nanoparticles (Figure 5G).

### 2.7 Dose‐Dependence of Tumor Efficacy

To test whether a greater AUC of the ABD‐CP‐PTX nanoparticles than CP‐PTX nanoparticles in tumors translates to a better therapeutic profile, we performed a tumor regression study in‐house at Duke University at a dose of 25 mg Kg^−1^ B.W. equivalent of PTX in two different mouse models: an orthotopic MDA‐MB‐231 and a s.c. PC3 model with ABD‐CP‐PTX, CP‐PTX, and nab‐paclitaxel (as a control) at the same equivalent PTX dose. CP‐PTX nanoparticles require a slow pump injection for dose of 25 mg Kg^−1^ B.W. equivalent of PTX, whereas ABD‐CP‐PTX can be administered as a bolus injection at that dose. This is because a colloidal suspension of ABD‐CP‐PTX in PBS at room temperature has a significantly lower viscosity (*P* = 0.02, unpaired *t*‐test) than CP‐PTX (Figure S10, Supporting Information).

**Figure 6:**
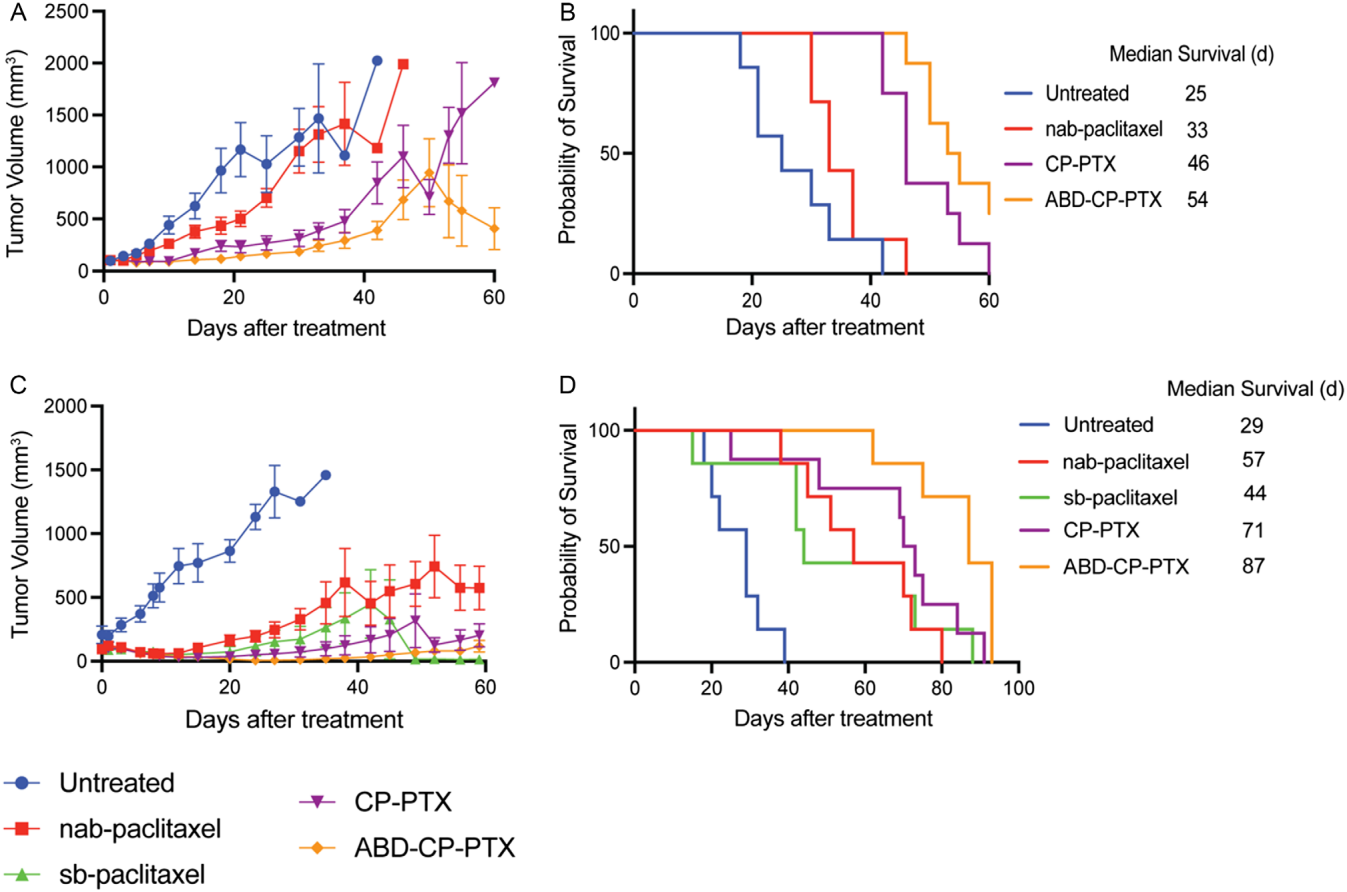
In vivo antitumor efficacy of ABD‐CP‐PTX. A,B) Orthotopic MDA‐MB‐231 breast tumor model. MDA‐MB‐231 cells were implanted in the mammary fat pad of female nude mice and allowed to grow to ≈100 mm^3^. C,D) Subcutaneous PC3 prostate cancer xenograft model. PC3 tumor cells were implanted s.c. in nude mice and allowed to grow to ≈100 mm^3^. Mice were then treated on day 0 with solvent‐based free‐PTX (sb‐paclitaxel), nab‐paclitaxel, CP‐PTX, and ABD‐CP‐PTX at 25 mg Kg^−1^ B.W. equivalent of PTX dose. (A,C) Tumor volume posttreatment. (B,D) Cumulative survival of mice up to day 60. Data represent mean ± SEM (*n* = 7–8 mice).

In the first model, mice bearing orthotopic MDA‐MB‐231 tumors with a size of ≈100 mm^3^ were infused i.v. with a single dose of nab‐paclitaxel, CP‐PTX, or ABD‐CP‐PTX at a dose of 25 mg Kg^−1^ B.W. equivalent of PTX on day 0 (**Figure** **6**A). The percentage body weight loss remained well below the 20% threshold cut‐off indicative of significant systemic toxicity, and no visible signs of distress were observed in any mice (Figure S11, Supporting Information) suggesting that this dose of all three formulations was well tolerated by the tumor‐bearing mice. On day 18 posttreatment, the mean tumor volume of untreated mice was 965 mm^3^ (*n* = 7). In comparison, the mean tumor volume for nab‐paclitaxel treated mice was 435 mm^3^ (*n* = 7), 242 mm^3^ for CP‐PTX treated mice (*n* = 8), and 117 mm^3^ for ABD‐CP‐PTX treated mice (*n* = 8). Compared to nab‐paclitaxel, the mean tumor volume was ≈1.8‐fold smaller for mice treated with CP‐PTX, but this difference is not statistically significant (Tukey's test; *P* = 0.05). In contrast, mice treated with ABD‐CP‐PTX nanoparticles showed a larger ≈3.7‐fold decrease in tumor volume relative to mice treated with nab‐paclitaxel and this difference is statistically significant (Tukey's test; *P* = 0.0002). Even on day 42 posttreatment, the ABD‐CP‐PTX treated group (*n* = 8) showed a 2.2‐fold and threefold lower tumor volume compared to the CP‐PTX (*P* = 0.001, Tukey's test) and nab‐paclitaxel (*P* = 0.01) treated group, respectively.

Treatment with a single dose of ABD‐CP‐PTX at the dose of 25 mg Kg^−1^ B.W. equivalent of PTX improved the median survival by 116% (Kaplan–Meier, Log‐rank test, *P* < 0.0001) whereas treatment with CP‐PTX nanoparticles and nab‐paclitaxel only improved survival by 84% (*P* = 0.0002) and 32% (*P* = 0.1), respectively, compared to untreated mice (Figure 6B). To put these results in perspective, the i.v. administration of free drug—sb‐paclitaxel—in MDA‐MB‐231 tumor‐bearing mice at a dose of 25 mg Kg^−1^ B.W. equivalent of PTX improved the median survival by 56% compared to untreated mice (Figure 6B). This demonstrates that at a lower dose such as 25 mg Kg^−1^ B.W. equivalent of PTX, nab‐paclitaxel is only as effective as the solvent‐based drug formulation (sb‐paclitaxel). Importantly, ABD‐CP‐PTX nanoparticles outperformed both CP‐PTX (by 22%, *P* < 0.05) and nab‐paclitaxel (by 53%, *P* < 0.005) in improving median survival.

In the second model, mice bearing s.c. PC3 tumors with a size of ≈100 mm^3^ were administered with a single i.v. dose of nab‐paclitaxel, CP‐PTX, or ABD‐CP‐PTX at 25 mg Kg^−1^ B.W. equivalent of PTX on day 0 (Figure 6C). On day 27 posttreatment, the mean tumor volume in untreated mice was 1392 mm^3^ (*n* = 7). In comparison, the mean tumor volume was 178 mm^3^ for sb‐paclitaxel (*n* = 7), 247 mm^3^ for nab‐paclitaxel (*n* = 7), 57 mm^3^ for CP‐PTX (*n* = 8), and only 8 mm^3^ for ABD‐CP‐PTX (*n* = 7), respectively. Compared to the nab‐paclitaxel group, the tumor volume was 1.6‐fold lower in the sb‐paclitaxel treated group (*P* = 0.7, ns, Tukey's test) and 4.3‐fold lower in the CP‐PTX group (*P* = 0.07, ns). Excitingly, mice in ABD‐CP‐PTX group had ≈30‐fold lower tumor volume compared to the nab‐paclitaxel group (*P* = 0.01). It is important to note that between week 2 and week 7, the tumor volume for ABD‐CP‐PTX was so small that it was difficult to reliably measure the tumor volume with calipers and hence the standard error of mean in some instances is greater than the mean, making the *P* value of 0.01 (statistically significant) higher than anticipated for such a large difference (≈30‐fold).

Treatment with a single dose of ABD‐CP‐PTX improved the median survival by 200% (Kaplan–Meier, Log‐rank test, *P* < 0.0005) whereas treatment with sb‐paclitaxel, CP‐PTX nanoparticles, and nab‐paclitaxel improved survival by 52% (*P* = 0.004), 144% (*P* = 0.001), and 97% (*P* = 0.0004), respectively, compared to untreated mice (Figure 6D). In some mice treated with sb‐paclitaxel, we observed shrinking of tumors 7 weeks posttreatment but the median survival was poor due to the sudden death of some mice without any signs of toxicity. We hypothesize that this is because PC3 tumors are highly metastatic, so that metastases in other vital organs led to death from vital organ failure rather than the primary tumor burden. The regression and survival data from the orthotopic and s.c. mouse tumor models collectively show that ABD‐CP‐PTX has a wider therapeutic window and better efficacy than CP‐PTX and nab‐paclitaxel, highlighting that this next‐generation albumin‐binding nanoparticle formulation is superior to the previous generation—CP‐PTX nanoparticles—as well nab‐paclitaxel for the treatment of solid tumors.

## 3 Discussion

Since its discovery, PTX has become a first‐line chemotherapeutic drug to treat a broad spectrum of solid tumors.^[^ ^41^, ^42^, ^43^, ^44^, ^45^ ^]^ However, the solvent used in the commercial formulation of PTX—a 1:1 solution of Kolliphor EL, formerly known as Cremophor EL and dehydrated ethanol—has several drawbacks. First, like all small molecule hydrophobic drugs, sb‐paclitaxel suffers from off‐target toxicity, poor plasma half‐life and systemic exposure, and requires multiple doses for treatment.^[^ ^6^, ^46^, ^47^ ^]^ Second, excipients in the sb‐paclitaxel formulation result in severe, sometimes fatal, hypersensitivity reactions in patients^[^ ^3^, ^4^, ^5^ ^]^ that necessitate pretreatment with corticosteroids and antihistamines.^[^ ^6^ ^]^ Third, Kolliphor EL can entrap PTX in solvent micelles, making the drug less available to enter tumors, thereby limiting its clinical efficacy.^[^ ^7^ ^]^ It also inhibits the binding of PTX to albumin and endothelial cells, potentially limiting the intratumoral accumulation of PTX.^[^ ^8^ ^]^ Finally, the administration of sb‐paclitaxel in the clinic is cumbersome, requiring a large infusion volume and hence a long infusion duration to minimize hypersensitivity reactions.^[^ ^5^ ^]^ In addition, the organic solvents cause leaching of plasticizers from PVC bags in the infusion setup that is commonly used in the clinic, so that it must be prepared and administered using a non‐PVC infusion system and requires in‐line filtration.^[^ ^48^, ^49^ ^]^

Over the last two decades, PTX nanoparticles have gained traction as an alternative to solvent‐based systems to circumvent the serious side effects of Cremophor EL and ethanol‐based free drug formulations,^[^ ^50^ ^]^ especially the severe allergic reactions caused by Cremophor EL. A diverse range of solvent‐free nanoformulations of PTX that improve aqueous solubility of the drug, protect the drug during formulation and storage, have been tested in preclinical settings, including Cynviloq,^[^ ^51^ ^]^ PICN,^[^ ^52^ ^]^ Lipusu,^[^ ^53^ ^]^ Liporaxel,^[^ ^54^ ^]^ Paclical,^[^ ^55^ ^]^ Opaxio,^[^ ^56^ ^]^ and Abraxane^[^ ^8^ ^]^ (**Table** **3**).

**Table 3:** Summary of preclinical efficacy data of the PTX‐formulations.

Formulation	Company/Source	Technology	Size [nm]	Drug release	Cumulative dose (Administration route)	Efficacy compared to PTX	Efficacy compared to Abraxane
Genexol‐PM currently known as Cynviloq	Sorrento Therapeutics	Polymeric micelle	20–50	Passive	180 mg kg^−1^ PTX eqv. (i.v.)	>2‐fold better tumor regression	Not reported
PICN	Sun Pharma Advanced	Polymeric‐lipid nanoparticle	100–150	Passive	No rodent data reported		
Lipusu	Luye Pharma	Liposomes	≈400	Passive	30 mg kg^−1^ PTX eqv. (i.v.)	Similar	Not reported
Liporaxel/DHP‐107	Daehwa Pharmaceutical		Not found	Passive	No rodent data found		
Paclical (Paccal‐vet)/Apealea	Oasmia Pharmaceutical	Micelle of retinoid compound XR‐17	20–60	Passive	No rodent data found		
Opaxio formerly known as Xyotax	Cell Therapeutics	Conjugate of poly‐glutamic acid	Not found	Active (Ester linkage)	350 mg kg^−1^ PTX eqv. (i.p.)	Not reported	1.5‐fold less at equimolar dose, 40% more at equitoxic dose
Abraxane	Celgene	Albumin‐based nanoparticle	≈130	Passive	30–47 mg kg^−1^ PTX eqv. (i.v.)	1.2‐ to 4.7‐fold better tumor regression	Not applicable
ABD‐CP‐PTX	This paper	Albumin‐binding nanoparticle	≈90	Active (Hydrazone linkage)	25–50 mg kg^−1^ PTX eqv. (i.v.)	3‐ to 20‐fold better tumor regression	1.8‐ to 30‐fold better tumor regression

These nanoparticles share a common feature: they completely avoid the use of Cremophor EL and ethanol. However, with the exception of Opaxio, none of these formulations have been compared to nab‐paclitaxel —the gold standard—in their preclinical development.^[^ ^57^ ^]^ Furthermore, the therapeutic outcomes of these formulations in a clinical setting have been suboptimal, with nab‐paclitaxel being the only formulation to receive FDA approval in 2005 under the brand name Abraxane.^[^ ^12^ ^]^ As of January 2024, clinicaltrial.gov reports ≈2800 clinical trials related to PTX nanoparticles, with an astonishing ≈96% of these trials focusing on nab‐paclitaxel.

Because nab‐paclitaxel is formulated with albumin, it is soluble in aqueous buffers. The improved efficacy of nab‐paclitaxel comes from its ability to hijack the endogenous albumin transport mechanism to cross the endothelial cell layer and enter tumors.^[^ ^58^ ^]^ Albumin, the most abundant serum protein, bypasses systemic clearance and degradation by the body's own innate mechanisms, so that it has an exceptionally long half‐life of 19 days in humans, and a similarly long half‐life in most animal species.^[^ ^58^ ^]^ The long half‐life of albumin is attributed to the neonatal Fc receptor (FcRn), a widely distributed intracellular receptor responsible for salvaging albumin from cellular catabolism.^[^ ^59^ ^]^ Because of its long plasma half‐life, albumin preferentially accumulates at sites of vascular leakiness and is internalized by rapidly growing, nutrient‐starved cancer cells. In a preclinical study, fourfold more nab‐paclitaxel was transported across endothelial cells than sb‐paclitaxel.^[^ ^8^ ^]^ In addition, nab‐paclitaxel enables administration of significantly higher doses of PTX over a shorter infusion time (30 min vs 3 h for sb‐paclitaxel), eliminates the need for premedications to prevent hypersensitivity reactions, and enhances long‐term stability of the PTX cargo.^[^ ^5^, ^8^, ^19^, ^60^, ^61^ ^]^

However, nab‐paclitaxel is far from ideal as a PTX formulation. First, the therapeutic outcomes of treatment with nab‐paclitaxel are far from satisfactory in the clinical setting. For instance, nab‐paclitaxel has a response rate of only 33% in breast cancer.^[^ ^11^, ^12^ ^]^ In addition, the incidence of grade‐3 sensory neuropathy in patients undergoing nab‐paclitaxel treatment is a significant concern, because neuropathy limits the dose of nab‐paclitaxel that can be safely administered.^[^ ^11^, ^13^, ^14^ ^]^ The suboptimal response rate of Abraxane in many cases is at least partially caused by its passive and slow drug release profile.^[^ ^10^ ^]^ Because PTX is entrapped via noncovalent interactions with albumin in Abraxane, its release is dictated by passive diffusion so that significant release of PTX takes up to a week.^[^ ^36^, ^62^, ^63^, ^64^ ^]^

Second, manufacturing of nab‐paclitaxel requires a complex emulsion‐evaporation crosslinking technique under high pressure.^[^ ^9^ ^]^ The removal of organic solvents is difficult, as organic solvents are packaged in the inner phase of the emulsion, so that it is expensive to reduce the level of organic solvent in the formulation.^[^ ^36^ ^]^ The high‐pressure homogenization technology and harsh formulation conditions can also result in a subtle conformational change of albumin that may result in adverse immunogenicity.^[^ ^65^ ^]^ To bypass these issues, Gao et al. recently developed a less complex technique to package paclitaxel into albumin.^[^ ^36^ ^]^

Third, nab‐paclitaxel uses exogenous albumin, which is produced by fractionation of plasma obtained from blood donors.^[^ ^66^, ^67^ ^]^ With the emergence of infectious diseases like COVID‐19, Zika, HIV, etc. blood‐derived products such as albumin are subjected to stringent screening of blood donors and require continuous development of new techniques to detect and inactivate emerging blood‐borne pathogens.^[^ ^68^, ^69^, ^70^ ^]^ Recombinant albumin is an alternative to address the issues with animal‐derived albumin. However, the high‐yield synthesis of recombinant albumin requires a specialized expression system that is not readily available.^[^ ^71^ ^]^ Clearly, an alternative solvent‐free stable nanoformulation of PTX that matches the favorable traits of nab‐paclitaxel but avoids the complex manufacturing steps and the use of exogenous albumin, or the difficulties in recombinant expression of human serum albumin is highly desirable.

To circumvent these issues, we have described herein a genetically engineered PTX‐loaded nanoparticle that packages the drug in the core of the nanoparticle through covalent attachment via an acid‐labile covalent bond and displays an albumin selective ligand—an ABD—on its surface that binds to the abundant endogenous albumin in plasma^[^ ^72^ ^]^ with nanomolar affinity after intravenous administration, thus creating an albumin corona on the nanoparticle. The albumin coating of the nanoparticle greatly prolongs the in vivo circulation time of the nanoparticle compared to unmodified nanoparticles that do not have an albumin corona. Each nanoparticle contains ≈130 PTX molecules that are released at low pH from the core of the nanoparticle upon cellular uptake (Scheme 1). The prolonged in vivo circulation of ABD‐CP‐PTX results in improved tumor accumulation compared to its predecessor—CP‐PTX—that does not bind albumin in systemic circulation. The improved tumor accumulation can be correlated to albumin mediated delivery of PTX, similar to nab‐paclitaxel, that targets tumors through a combination of mechanisms that enhance its delivery and efficacy compared to conventional PTX formulations: 1) tumors typically have leaky blood vessels and poor lymphatic drainage, which allow nanoparticles to accumulate more in tumor tissues than in normal tissues—the so‐called “Enhanced Permeability and Retention (EPR)” effect; 2) albumin can bind to receptors on endothelial cells of blood vessels, such as gp60, and transport drugs across the endothelial barrier through a process called transcytosis^[^ ^8^, ^73^ ^]^; and 3) tumor cells often overexpress secreted protein acidic and rich in cysteine (SPARC),^[^ ^74^ ^]^ a protein that binds albumin. Similar to nab‐paclitaxel, albumin‐bound ABD‐CP‐PTX nanoparticle can exploit this by binding to SPARC, leading to increased retention and uptake of the drug by the tumor cells.

The improved tumor accumulation for ABD‐CP‐PTX nanoparticles translates into at least a twofold wider therapeutic window compared to nab‐paclitaxel in two different murine solid tumor models. This expansion of the therapeutic window holds significant relevance for clinical application. Mice, unlike humans, exhibit greater resilience to the physiological stresses induced by chemotherapy at levels that would likely lead to severe systemic side effects in humans. Therefore, reducing the dosage necessary for achieving a therapeutic outcome below the MTD in mice bodes well for the clinical advancement of ABD‐CP‐PTX. These results also suggest that ABD‐CP‐PTX may overcome the dose‐limiting toxicity seen with nab‐paclitaxel at the doses required for therapeutic benefit in humans.^[^ ^75^ ^]^ The lower viscosity of the highly concentrated ABD‐CP‐PTX solution compared to CP‐PTX may enhance “syringeability” and simplify manufacturing. Assuming all other factors are equal, several interrelated parameters could explain the observed viscosity differences between CP‐PTX and ABD‐CP‐PTX formulations. These include charge, molecular shape, adsorbed ions on the polypeptides, self‐assembly characteristics (such as size, aggregation number, and hierarchy at high concentrations), ionic strength, and shear rate.^[^ ^76^ ^]^ To fully understand these differences, a comprehensive rheological response of the polypeptide solutions needs to be evaluated in future studies using rheological measurements like steady shear, small and large amplitude oscillatory shear, and creep/relaxation tests. Finally, a comprehensive evaluation of both chronic and acute toxicity, including assessments of hematology, blood biochemistry, tissue pathology, and genotoxicity, is necessary to support the clinical translation of ABD‐CP‐PTX. These evaluations are planned for subsequent studies.

## 4 Conclusion

The four most important findings of this study are as follows. First, ABD‐CP‐PTX nanoparticles can be lyophilized and stored at subzero temperature for at least one year without any excipients and can be reconstituted on‐demand in aqueous buffer at high concentration, thus greatly simplifying the overall formulation process. Second, the formulation completely avoids use of exogenous—blood derived—albumin. Third, albumin‐binding ABD‐CP‐PTX nanoparticles bind to mouse and human serum albumin with nanomolar affinity, and when injected intravenously, improve the bioavailability of PTX by 2.7‐fold, resulting in improved tumor accumulation by twofold compared to its first‐generation parent—CP‐PTX nanoparticles—that do not bind albumin. Fourth, the greater tumor accumulation of the ABD‐CP‐PTX nanoparticles translates to greater efficacy across a wider therapeutic window compared to nab‐paclitaxel in two different mouse models. Given that ABD‐CP‐PTX exhibits superior efficacy compared to clinically approved PTX formulations, we suggest that it is a compelling option for further development as an alternative to nab‐paclitaxel, the current “gold standard” for treatment of solid tumors with PTX.

## 5 Experimental Section

Material's source, methods of synthesis and characterization, and experimental methods are provided in the Supporting Information (SI).

## Conflict of Interest

The authors declare no conflict of interest.

## Supporting information

Supplementary Material

## Data Availability

The data that support the findings of this study are available from the corresponding author upon reasonable request.
