# Supplementary material for: Preclinical Development of a Genetically Engineered Albumin‐Binding Nanoparticle of Paclitaxel
Source: Small Sci. 2024 Sep 25;4(11):2400153. doi: 10.1002/smsc.202400153 (PMC11934972; doi:10.1002/smsc.202400153)
Supplement: Supplementary file 1 — Supplementary Material [file SMSC-4-2400153-s001.pdf]

Preclinical Development of a Genetically Engineered Albumin-Binding Nanoparticle of Paclitaxel

Soumen Saha[#], Samagya Banskota[#], Parisa Yousefpour, Jeffrey L. Schaal, Nikita Zakharov, Jianqiao Liu, Michael Dzuricky, Ziwei He, Stefan Roberts, Xinghai Li, and Ashutosh Chilkoti*

[#]Equal contributing authors

S. Saha, S. Banskota, P. Yousefpour, JL. Schaal, N. Zakharov, J. Liu, M. Dzuricky, Z. He, S. Roberts, X. Li, A. Chilkoti: Department of Biomedical Engineering, Pratt School of Engineering, Duke University, Durham, North Carolina 27708, United States

Email: chilkoti@duke.edu

Keywords: Preclinical drug development, recombinant nanoparticle, elastin-like polypeptide, albumin binding domain, nab-paclitaxel, cancer

Supplementary Tables

Table S1: One letter abbreviated amino acid sequence of ABD and CP

**ABD**	LAEAKVLANRELDKYGVSDFYKRLINKAKTVEGVEALKLHILAALP
**CP**	(VPGXG)(CGG)$_8$WP; X = V:G:A 7:8:1

Table S2: Calculated thermodynamic parameters of CP-PTX from isothermal calorimetry

Thermodynamic parameters / Conjugates-albumin interaction	MSA		HSA	
	CP	CP-PTX	CP	CP-PTX
Binding stoichiometry (N)	0.85	NA	1.46	NA
Dissociation Constant (K_D, nM)	3.5	NA	4.50	NA

The binding and thermodynamic parameters —binding constant (KD) and number of binding sites (N)— were computed by non-linear curve fitting of the data to a single site binding model using the Origin Lab software provided with the VP-ITC calorimeter.

Table S3: Murine pharmacokinetic parameters of ABD-CP-PTX and CP-PTX

	5 mg/Kg		25 mg/Kg		50 mg/Kg	
	ABD-CP-PTX	CP-PTX	ABD-CP-PTX	CP-PTX	ABD-CP-PTX	CP-PTX
Half-life (h)	5.5 ± 0.9	3.0 ± 0.7	13.1 ± 1.1	14.0 ± 1.5	14.0 ± 0.8	14.9 ± 1.2
Clearance (mL/h)	5.0 ± 2.0	14 ± 4.0	3.0 ± 1.0	3.0 ± 1.0	2.0 ± 1.0	1.0 ± 0.4
AUC (μM.h)	1458.1 ± 260.4	292.7 ± 43.8	7796.7 ± 1061.7	2873.4 ± 689.1	8479.2 ± 771.9	6027.9 ± 887.8
Mean retention time (h)	7.8 ± 1.3	4.1 ± 0.8	18.6 ± 1.7	19.9 ± 2.1	19.7 ± 1.0	19.7 ± 1.0
Volume of distribution at steady state (mL)	1.0 ± 0.1	3.0 ± 0.6	1.0 ± 0.1	4.0 ± 0.5	1.0 ± 0.1	1.0 ± 0.1

[a]Phamacokinetic (PK) parameters were assessed using PK solver software.

Supplementary Figures

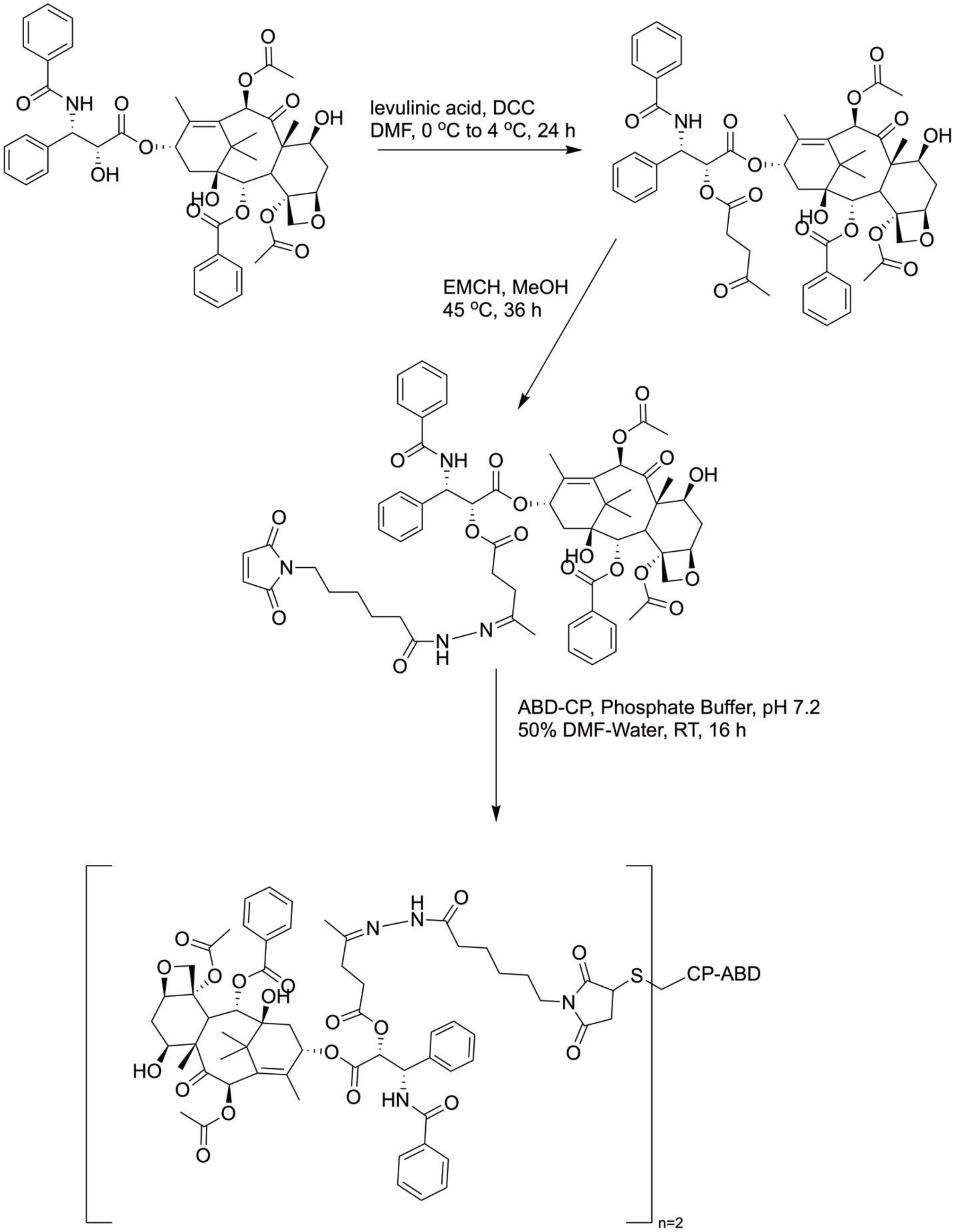

Figure S1: Synthesis scheme for ABD-CP-PTX.

A)

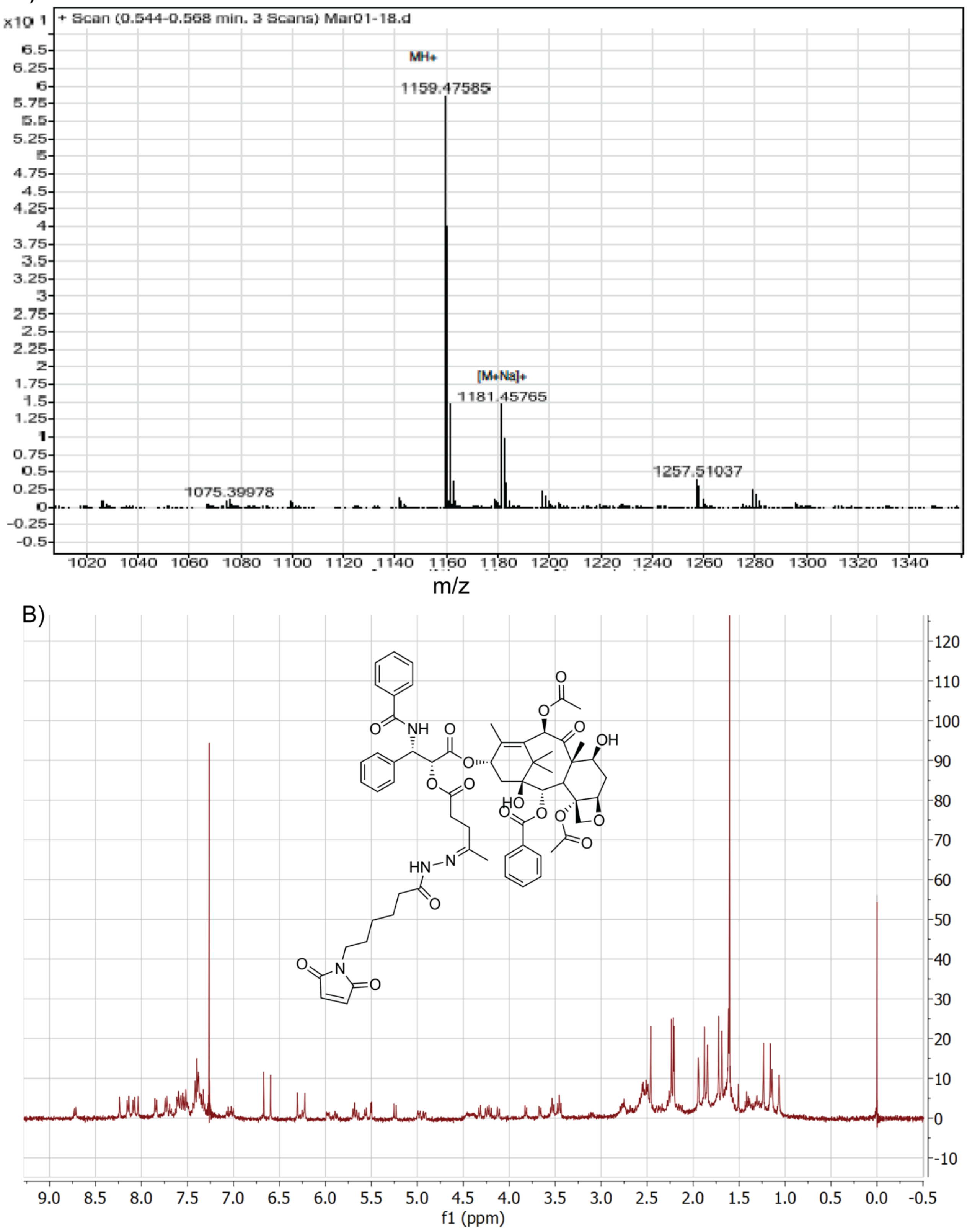

Figure S2: ESI-MS (A) and proton NMR (B) spectroscopy of the paclitaxel prodrug.

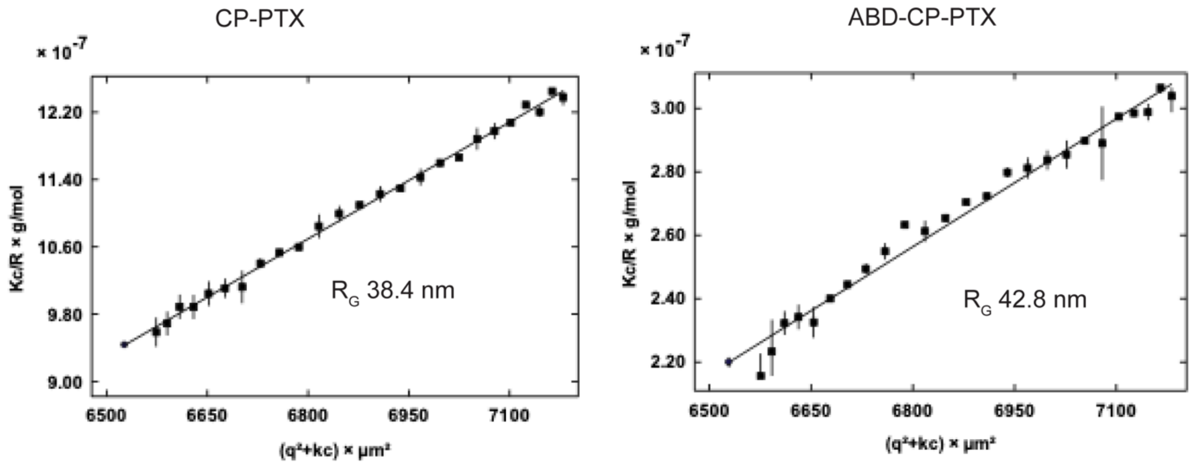

Figure S3: Partial Zimm plot (Kc/R vs q2+kc) of CP-PTX and ABD-CP-PTX from Static Light Scattering. The ALV/Dynamic and Static FIT and PLOT software were used to calculate the partial Zimm plots and were used to determine the Rg and MW of the nanoparticles. The aggregation number (Nagg) of each PTX-loaded nanoparticle was obtained by dividing the MW of the nanoparticles obtained from the partial Zimm plot by the MW of unimers of each PTX conjugate.

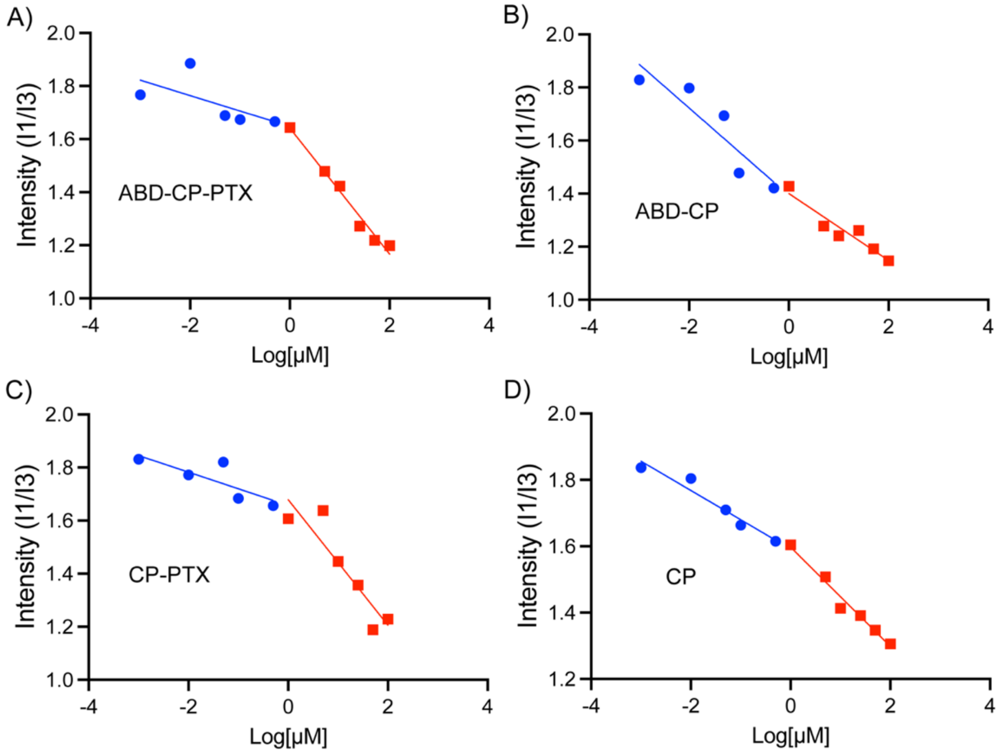

Figure S4: Determination of critical aggregation concentration (CAC) by pyrene fluorescence assay. Fluorescence intensity was measured as a function of concentration of free polypeptides (CP and ABD-CP) and PTX conjugates. The solid lines represent the best linear fit for the two regions. CAC was calculated from the X co-ordinate of the intersection of the two linear fits using PRISM.

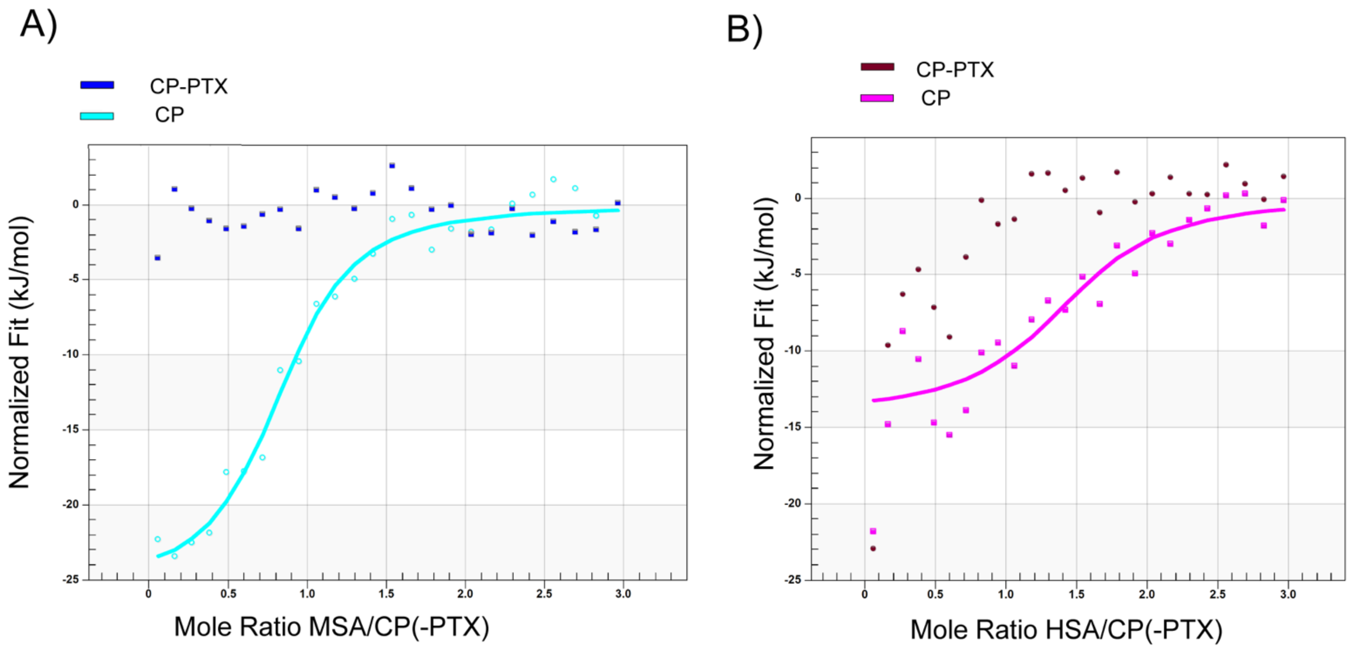

Figure S5: Isothermal calorimetric titration of albumin binding of CP and CP-PTX. The experiments were performed in PBS (pH 7.4) at 37 °C. The solid line indicates the best-fit binding of the binding isotherm. MSA (A) or HSA (B) at 500 μM was titrated into the sample cell containing 50 μM of CP-PTX. The integrated heat data were fit to a single site binding model and the binding stoichiometry (N), and dissociation constant (K_D) were calculated, as shown in **Table 2**.

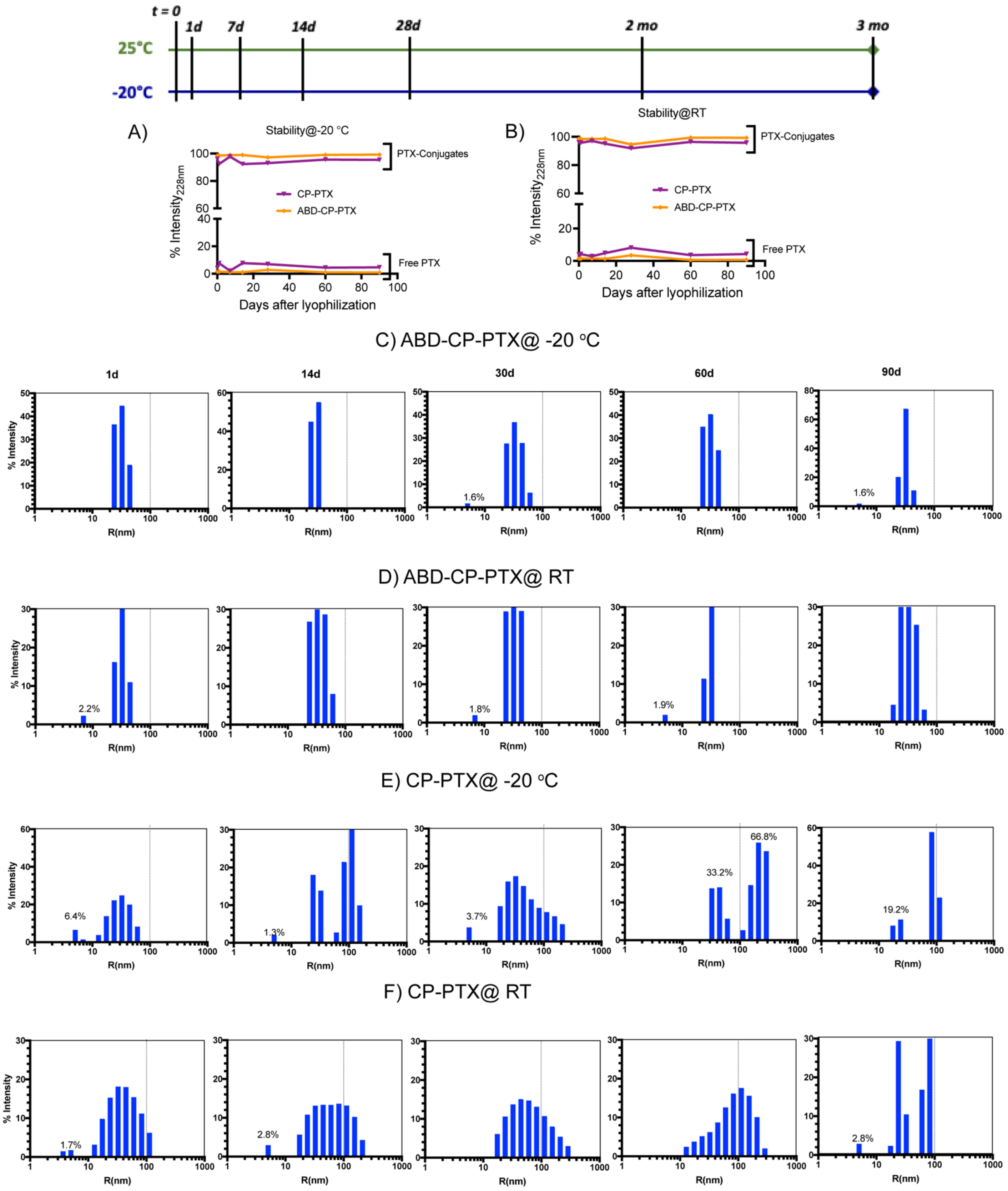

Figure S6: Long-term stability analysis of ABD-CP-PTX. Lyophilized conjugates were stored at -20 °C or RT. At indicated time points samples were thawed and reconstituted in PBS and analyzed by SEC-HPLC (A-B) and DLS (C-F). Free drug from the conjugate was separated on the SEC column and detected using a UV-

detector at 228 nm. Less than 5% drug got released. DLS analysis revealed only lyophilized ABD-CP-PTX can be reconstituted as a monodisperse solution (C-D) even after 3 months while CP-PTX showed higher order aggregates (Rh > 100 nm) as soon as 14 days (E-F) and disintegration of nanoparticles by 90 days.

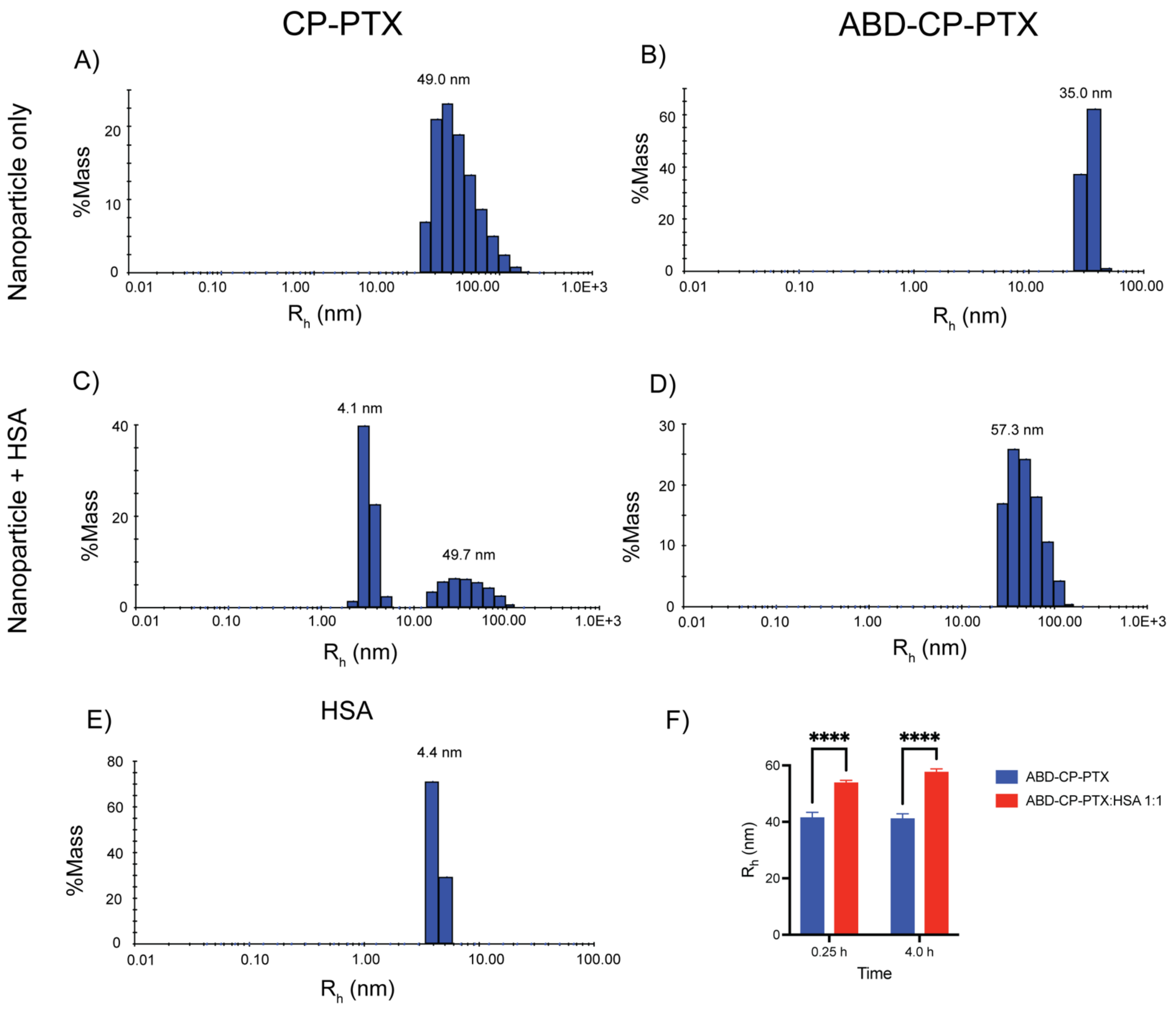

Figure S7: Long-term albumin-binding ability of ABD-CP-PTX. Lyophilized conjugates were stored at -80 °C for one year. Samples were thawed and reconstituted in PBS—without HSA or with equal mol of HSA— and analyzed by DLS at physiological temperature and pH. DLS analysis revealed a single micellar population for native nanoparticles (A-B). However, size of ABD-CP-PTX nanoparticle increases significantly in presence of albumin due to efficient binding whereas the size remains virtually same for CP-PTX nanoparticle (C-D). Regularization fit of the scattering data indicates presence of two population (C) for CP-PTX suggesting their lack of affinity towards albumin. The smaller particle comes from the unbound albumin which matches closely with the size of free albumin (E). In contrast, ABD-CP-PTX incubated with albumin shows only one population

(D). Moreover, this increase in size remains the same after 4 h of incubation (F) indicating strong affinity of ABD-CP-PTX towards albumin and their stability.

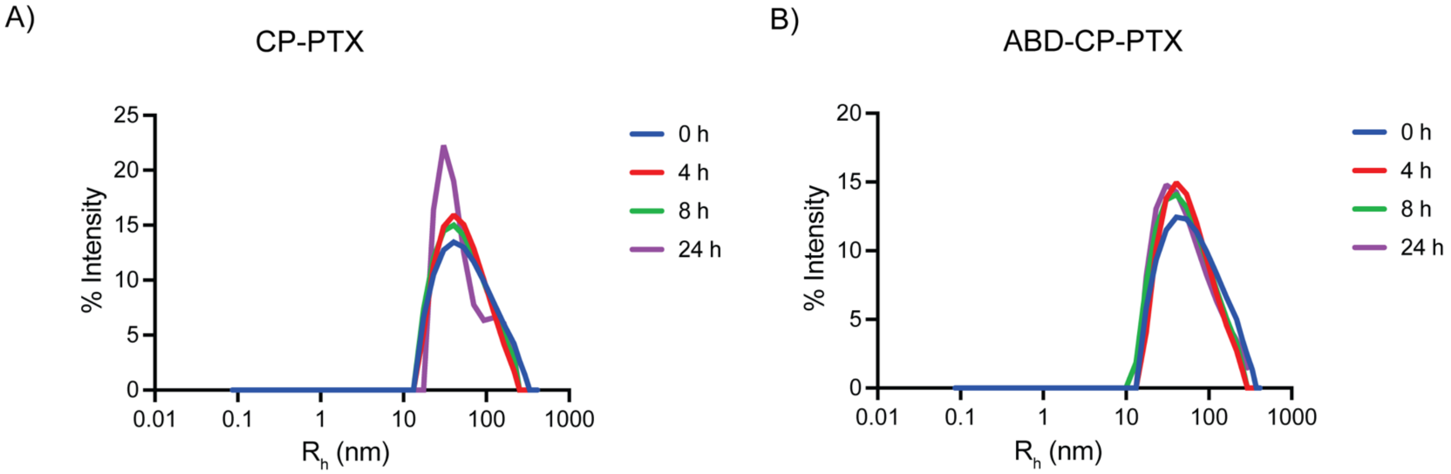

Figure S8: Short-term stability of CP-PTX and ABD-CP-PTX and nanoparticles at room temperature. Freshly prepared lyophilized samples were dissolved into PBS and incubated at room temperature (RT). At the indicated timepoint, the hydrodynamic radius (R_h) was measured by dynamic light scattering (DLS). Reconstitution of lyophilized ABD-CP-PTX in PBS resulted into monodisperse nanoparticles that remained stable for at least 24 h at RT with no sign of higher order aggregates. In contrast, CP-PTX nanoparticles showed appearance of aggregates after 8 h.

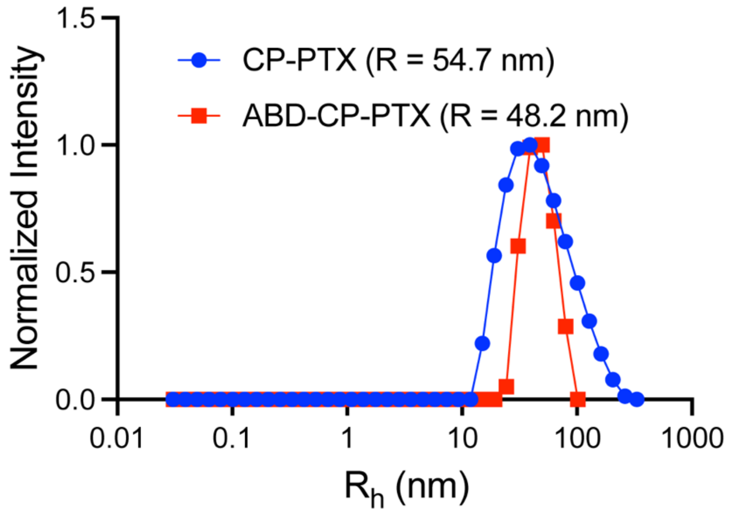

Figure S9: DLS analysis of cyanine labeled PTX nanoparticles. Attachment of the maleimide modified cyanine to the PTX conjugate did not change the hydrodynamic radius (R_h) significantly compared to unlabeled drug conjugates (**Figure S6**).

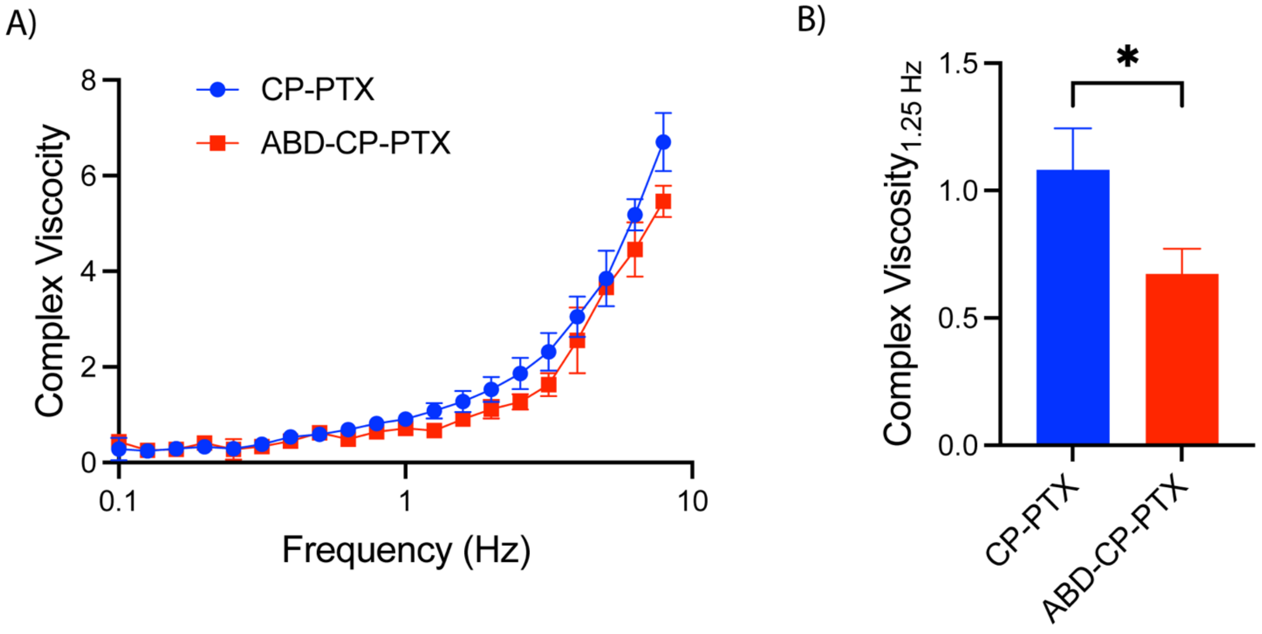

Figure S10: Complex viscosity measurement of PTX nanoparticles. Viscosity of saturated ABD-CP-PTX and CP-PTX in PBS was measured on a Kinexus Pro rheometer (Malvern) by a frequency sweep between 0.1-100 Hz with one unit of strain. A significant drop in complex viscosity was observed for ABD-CP-PTX relative to CP-PTX. Data was acquired for each sample in triplicate.

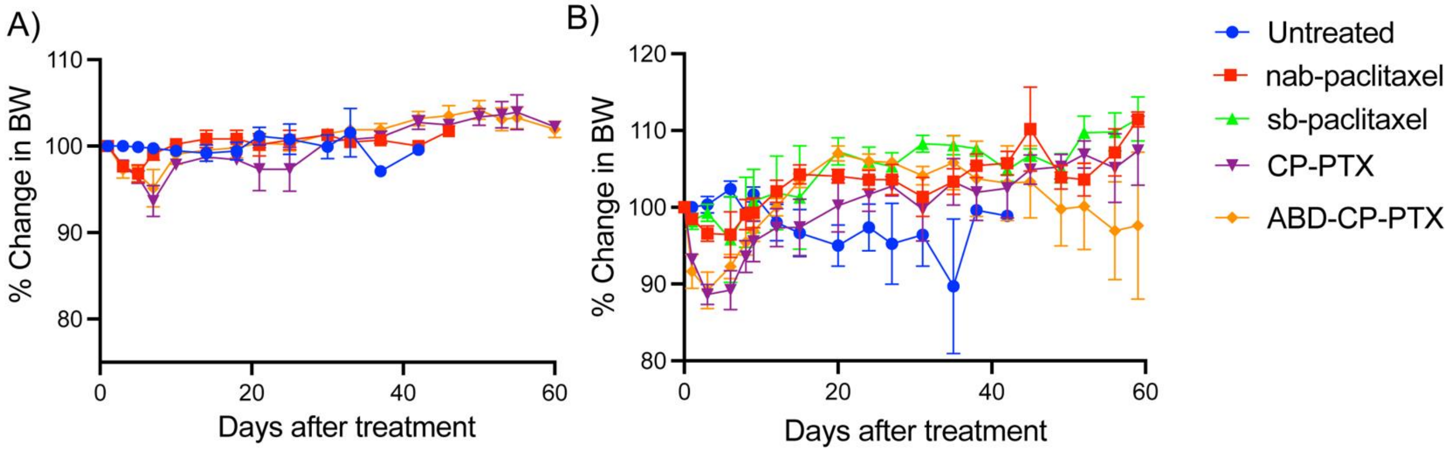

Figure S11: Change in %Body weight post treatment. (A) Mice bearing orthotopic MDA-MB-231 tumors and (B) mice bearing s.c. PC3 tumors. The % body weight loss remained well below the threshold 20% cut-off for significant systemic toxicity.

Materials and methods

Materials. pET-24+ plasmid and BL21(DE3) E. coli cells was procured from Novagen Inc (Madison, WI). 5'-phosphorylated oligonucleotides encoding ABD were purchased from Integrated DNA technologies (Coralville, IA). DNA mini-prep and gel purification kits were purchased from Qiagen Inc. (Germantown, MD). Restriction endonuceases, T4 DNA ligase, and Quick ligase, were purchased from New England Biolabs (Ipswich, MA). Bacterial cultures were grown in 2X YT media that contains sodium chloride (5 g L-1; Alfa Aesar, Ward Hill, MA), tryptone (16 g L-1, Becton, Dickinson and Co., Franklin Lakes, NJ), and yeast extract (10 g L-1, Becton, Dickinson and Co., Franklin Lakes, NJ). Kanamycin sulfate, human serum albumin (HSA) and mouse serum albumin (MSA) were purchased from EMD Millipore (Billerica, MA). Protein expression was induced with isopropyl β-d-1-thiogalactopyranoside (IPTG) from Gold Biotechnology (St. Louis, MO). All salts used for protein purification were purchased from Alfa Aesar (Ward Hill, MA). Tris(2-carboxyethyl)phosphine (TCEP) hydrochloride was purchased from Biosynth International (Itasca, IL). Detoxi-Gel resin was purchased from Thermo Fisher Scientific (Waltham, MA). Any kD TGX gels, protein molecular weight marker (Precision Plus Protein unstained standards), and Laemmli's sample buffer were purchased from Bio-Rad Laboratories (Hercules, CA). Paclitaxel was purchased from Ark Pharm (Arlington Heights, IL), levulinic acid was purchased from TCI America (Portland, OR), 4-(dimethylamino)pyridine (DMAP) was purchased from Alfa Aesar (Haverhill, MA), and N,N'-dicyclohexylcarbodiimide was purchased from Sigma Aldrich (St. Louis, MO). N-ε-maleimidocaproic acid hydrazide (EMCH) was purchased from Thermo Fisher Scientific (Hampton, MA). Amicon Ultra-15 Amicon ultrafiltration spin columns (10-30 kDa molecular weight cut-off (MWCO)) were purchased from EMD Millipore (Billerica, MA). TLC silica plate (25 x 75 mm) was purchased from Agela Technologies (Torrance, CA), and silica gel (70-200 mesh) was purchased from Alfa Aesar (Haverhill, MA). Anhydrous methanol and dimethylformamide (DMF) were purchased from Sigma Aldrich (St. Louis, MO), and chloroform, ethyl ether, triethylamine, and acetonitrile were purchased from VWR BDH Analytical Chemicals (Radnor, PA).

Synthesis and Purification of CP and ABD-CP. The chimeric peptide (CP) consists of the sequence SKGPG(XGVPG)$_{160}$(CGG)$_8$WP (single amino-acid codes) where the guest residue X = V:G:A is in a 1:7:8 ratio, and was recombinantly synthesized and purified as described before.[1] The albumin binding domain (ABD) used herein is an engineered variant of a native ABD that has a higher affinity for albumin compared with the wild-type domain.[2–4] The oligonucleotides encoding the ABD was designed to contain sticky overhangs to allow their ligation into the vector containing the CP gene. A modified pET-24(+) plasmid encoding the CP gene was used and was isolated from NEB 5-alpha cells that were previously transformed with the plasmid using a QIAprepTM spin miniprep kit (Qiagen; Germantown, MD). The plasmid was digested with BseRI to create sticky ends at the 5' end of the CP gene. The two ABD-encoding singe stranded oligonucleotides were annealed and ligated into the BseRI-digested plasmid using T4 DNA Quick Ligase for 10 min at room temperature, and were then transformed into chemically competent NEB 5-alpha cells. Transformants were

selected on TB-agar plates containing 100 µg/mL kanamycin, and positives were submitted for bidirectional DNA sequencing. To express ABD-CP, transformed BL21(DE3) cells were inoculated in 50 mL 2X YT media containing kanamycin (45 µg.mL-1) at 250 rpm and 37°C for 16 h. The bacterial culture was then pelleted, resuspended in PBS, and used to inoculate 1 L of 2X YT media that were grown under the same conditions. Cells were induced with 1 mM IPTG at mid-log phase and incubated at 37 °C overnight with shaking. Cells were harvested, re-suspended in 2.5 mL PBS per L culture, and disrupted by ultrasonication (Misonix; Farmingdale, NY). Nucleic acid contaminants were pelleted by adding polyethyleneimine (20% v/v, 2 mL per liter culture) and centrifuging at 14,000 rpm and 4 °C for 20 min. ABD-CP was purified from the supernatant using two cycles of inverse transition cycling (ITC).[5] In the first cycle, the supernatant was incubated for 10 min at 50 °C, followed by 10 min on ice with occasional vortexing, and was then centrifuged at 14,000 rpm for 15 min at 4 °C. The supernatant was supplemented with sodium chloride to a final concentration of 2.5 M and was heated to 50 °C to trigger the phase transition. The insoluble coacervate, enriched with the ABD-CP, was collected by centrifugation at 14,000 rpm for 15 min at 37 °C. The pellet was resolubilized in 30 mM tris-carboxyethyl phosphine hydrochloride (TCEP-HCl), pH 7.0 (Thermo Fisher Scientific, Waltham, MA) by incubating in an ice-bath with occasional vortexing. A second ITC was performed similarly but with a final concentration of 0.5 M sodium chloride. The purity of ABD-CP was determined by SDS-PAGE. The purified product was then dialyzed against water, lyophilized, and stored at - 20 °C until further use.

Synthesis of Drug Conjugates. To conjugate paclitaxel (PTX) to the cysteine0rich drug attachment domain (DAD) of CP and ABD-CP, a maleimide moiety was first appended to PTX by a multi-step reaction as previously described with following modifications.[6] Briefly, the 2'-OH group of PTX was functionalized with levulinic acid (Lev) in the presence of DCC and DMAP. The conjugate (PTX-Lev) was purified using column chromatography, followed by its condensation with EMCH. The linker-modified PTX (PTX-Lev-EMCH) was purified by column chromatography that was preincubated and washed with 7.5% triethylamine-MeOH. The pure compound was eluted with 1.1% MeOH-$CHCl_3$ (yield 60%). The linker-modified PTX (PTX-Lev-EMCH, 0.3 mmol) was then conjugated to the thiol groups (0.06 mmol) in the Cys-rich DAD domain of the CP or ABD-CP. The reaction was carried out in 2:1 (v/v) DMF:phosphate buffer (50 mM, pH 7.4). After 24 h, excess reagents were removed by diluting the reaction to 3% DMF, followed by sequential ultrafiltration (Amicon Ultra-15, MWCO: 10 kDa) with 25% acetonitrile-PBS, and ultrapure water (pH adjusted to 7.4 by ammonium bicarbonate). To scale-up the conjugate to the gram scale with tangential flow filtration (TFF, Repligen), the reaction mixture was directly dia-filtered with 50% DMF-PBS (10 dia-volume) using a 30 kDa MWCO membrane filter, followed by water (pH adjusted to 7.4 with ammonium bicarbonate) and concentrated. The concentrated solution was then freeze dried (Labconco FreeZone, Kansas City, MO) and stored at - 80 °C until further use.

Size Exclusion Chromatography (SEC). Purity of the CP-PTX and ABD-CP-PTX was assessed using size exclusion chromatography (SEC). Samples were run on a Shodex OHPak SB-804 SEC column on a Shimadzu Prominence High Performance Liquid Chromatography (HPLC) system with an isocratic flow of 0.5 mL/min of

70% PBS and 30% acetonitrile. The HPLC data was quantified using the integrated area under the curve (AUC) at an absorbance of 228 nm that corresponds to the maximum absorbance of PTX.

MALDI-TOFMS : The drug attachment ratio of PTX to CP or ABD-CP was determined by matrix-assisted laser desorption/ionization time-of-flight mass spectrometry (MALDI-TOFMS). Briefly, 100 µM of samples were mixed with a saturated sinapinic acid matrix supplemented with 1% TFA at a ratio of 1:5. 2 µl of the sample mixed with the matrix were deposited onto a ground steel target plate and dried at room temperature. Samples were run on a Bruker Autoflex Speed LRF MALDI-TOF system calibrated against ProteoMass™ Aldolase (Sigma Aldrich, St. Louis, MO). The mass difference between the native polypeptide (CP or ABD-CP) and PTX-conjugated polypeptide was divided by the MW of PTX-Lev-EMCH (1158 Da) to calculate the number of PTX molecules conjugated per polypeptide chain.

SDS- and Native-PAGE. The purity of the ABD-CP and CP was assessed by SDS-PAGE. Native-PAGE was used to visualize the interaction of the ABD-CP unimer and ABD-CP-PTX nanoparticles with albumin. For SDS-PAGE, approximately 20 µg sample peptides were loaded in a 10 µL volume on a 20% Mini-PROTEAN® TGX Stain-Free™ gel (BioRad, Hercules, CA). Electrophoresis was performed in Tris-glycine buffer (2.5 mM Tris-base, 19.2 mM glycine, pH 8.3) containing 0.1% SDS at 180 V for 30 min. For native-PAGE, ABD-CP monomer and ABD-CP-PTX nanoparticles were mixed at a 1:1 molar basis with human serum albumin (HSA) or mouse serum albumin (MSA) and were loaded (20 µg per sample) on the gel. Native gel electrophoresis was run under the same conditions and with the same stacking gel and running buffer as SDS-PAGE, except that no SDS was used. The gels were visualized using the Gel Doc® imaging system (Bio-Rad, Hercules, CA).

Isothermal Titration Calorimetry. The interaction of ABD-CP and CP unimers, and the ABD-CP-PTX and CP-PTX nanoparticles, with HSA and MSA were measured by isothermal titration calorimetry (ITC) (VP-ITC, MicroCal LLC, Northampton, MA,). Aliquots of 5 µL of 500 µM HSA or MSA in PBS were titrated via a 250 µL syringe at intervals of 5 min into 1.435 mL of a 50 µM solution of the unimers or nanoparticles, with stirring at 300 rpm at 37 °C. The background heat of dilution was measured in a separate experiment by titrating 500 µM HSA or MSA (in the syringe) into PBS (in the cell) under the same injection conditions. The heat of dilution was subtracted from the titration curves and the binding and thermodynamic parameters —binding constant (K_D) and number of binding sites (N)— were computed by non-linear curve fitting of the data to a single site binding model using the Origin Lab software supplied with the VP-ITC calorimeter.

Temperature-Programmed Turbidimetry. Thermal profiles of CP, ABD-CP, and their PTX conjugates were determined by measuring the measuring the turbidity at a wavelength of 350 nm on a UV-vis spectrophotometer equipped with a temperature controller (Cary 300, Varian Instruments, Walnut Creek, CA) by raising the temperature at 1 °C/min over a range of concentrations (5, 10, 25, 50, and 100 µM) in PBS. The T_t was defined as the temperature at which the first derivative of the optical density with respect to solution temperature was the maximum.

Light Scattering: The hydrodynamic radius (R_h) and polydispersity (P_d) of CP- PTX and ABD-CP-PTX nanoparticles were determined by dynamic light scattering (DLS) using a temperature-programmed DynaPro microsampler (Wyatt Technology, Santa Barbara, CA). Samples were prepared in PBS and filtered (0.45 µm Millex-GV filters). At least five acquisitions were taken at 37 °C, and the data were analyzed by a regularization fit of the autocorrelation function using DYNAMICS v7 software (Wyatt technology). Static light scattering (SLS) measurements were performed on an ALV/CGS-3 goniometer system (Langen, Germany) to determine the radius of gyration (R_g) and the aggregation number (N_{agg}) of the nanoparticles. The nanoparticles were dissolved in PBS at a concentration of 5 µM and filtered through 0.45 µm Millex-GV filters into a 10 mm diameter disposable borosilicate glass tube (Fisher). SLS was carried out at 37 °C between 30°- 150° angles at 5° increments, where the measurement at each angle consisted of 3 runs for 15 seconds. The differential refractive index (dn/dc) was quantified by determining the refractive index at five dilutions (3-20 µM) using an Abbemat 500 refractometer (Anton Paar, Graz, Austria). ALV/Dynamic and Static FIT and PLOT software was used to obtain the partial Zimm plots to determine the R_g and MW of the nanoparticles. The N_{agg} was obtained by dividing the MW of the nanoparticles by the MW of the CP-PTX or ABD-CP-PTX conjugates obtained from MALDI.

Cryogenic Transmission Electron Microscopy (Cryo-TEM). Cryo-TEM images of the PTX nanoparticles were acquired at the Shared Materials Instrumentation Facility at Duke University (Durham, NC). Lacey holey carbon grids (Ted Pella, Redding, CA) were glow discharged on a PELCO EasiGlow Cleaning System (Ted Pella, Redding, CA). 3 µl of each sample at a concentration of 25 µM was deposited onto the grid, blotted for 3 s with an offset of -3 mm, and vitrified in liquid ethane using a Vitrobot Mark III (FEI, Eindhoven, Netherlands). To prevent sample evaporation prior to vitrification, the sample chamber was kept at 15 °C and 100% relative humidity. Grids were then transferred to a Gatan 626 cryoholder (Gatan, Pleasanton, CA) and imaged on a FEI Tecnai G2 Twin TEM instrument (FEI, Eindhoven, Netherlands)), operating under low-voltage conditions at 80 keV. The feature radius (R_{TEM}) was measured manually in ImageJ for at least 30 particles.

Pyrene Assay: The critical aggregation concentration (CAC) of CP-PTX and ABD-CP-PTX nanoparticles were assessed by fluorescence spectroscopy using pyrene as a fluorescent probe of the local hydrophobicity.[7] 12 mM of a pyrene stock solution was first prepared in ethanol and sonicated for 10 min at room temperature. It was then diluted to 20,000X in PBS by sonication. The native polypeptides and PTX-conjugates were then dissolved in the diluted pyrene solution in PBS at a concentration ranging from 1 nm to 100 µM. The fluorescence emission spectrum of each sample was then recorded on a Cary Eclipse fluorescence spectrometer equipped with a Xenon flash lamp (Varian Instruments, Palo Alto, CA) at Ex: 334 nm and Em: 360-380 nm. The ratio of the first fluorescence emission peak at 370-373 nm (I1) and the third peak at 381-384 nm (I3) were measured as a function of log-concentration. Two linear equations were fit to the obtained data and the CAC was calculated as the concentration at the intersection point of the two linear fits.

pH-Dependent Drug Release. To study the pH dependent drug release of PTX from ABD-CP-PTX and CP-PTX nanoparticles, samples were incubated at 37 °C in two different pH buffers: 100 mM sodium acetate pH

5.3 (endosomal) and sodium phosphate pH 7.4 at final polypeptide concentration of 30 µM (~60 µM of PTX equivalent). Samples were incubated for specified times —5 min, 30 min, 1 h, 2 h, 4 h, 6 h, 12 h, 24 h and 48 h— and at each time point, samples were quenched by dilution of samples in acetonitrile and phosphate buffer at pH 7.4 such that the final acetonitrile concentration was 30% and the pH was greater than 7.0 to stop hydrolysis. The samples were frozen immediately on dry ice. 50 µL of an aliquot at each time point was injected into an LC10 HPLC (Shimadzu Scientific Instruments; Columbia, MD) with a Shodex OHPak SB-804 column (New York, NY) using 30% PBS:acetonitrile (v/v) as the mobile phase at an isocratic flow rate of 0.5 mL/min. Eluting peaks were detected with a UV-vis detector, set at 228 nm corresponding to absorbance maxima of PTX. The percentage of unbound PTX in samples was calculated by integrating the chromatogram to determine the percentage peak area corresponding to unbound PTX. The cumulative percent released drug at each time point was defined as the concentration of unbound PTX at that time point divided the by total PTX (60 µM) concentration times 100.

Viscosity Measurement. Saturated samples of ABD-CP-PTX and CP-PTX (1.7 mM) were prepared in PBS and allowed to equilibrate at 4 °C. Measurements were taken on a Kinexus Pro rheometer (Malvern) using a Peltier heating element and a 10 mm parallel plate geometry. Samples were enclosed in a humidified environment to prevent drying during rheology. 30 µL of samples were loaded onto the lower portion of the geometry set at 4 °C. The upper portion of the geometry was lowered to 0.5 mm, and the instrument was subsequently heated to the experimental temperature (25 °C) and allowed to equilibrate for 30 min. To account for volume contraction, samples were run with a normal force control of 0.1 N—determined to be the optimal normal force to maintain geometry contact without sample deformation. Frequency sweeps were used between 0.1-100 Hz with one unit of strain. Data was acquired for each sample in triplicate.

Cell Culture. The human PC-3M-luc-C6 cells were purchased from Perkin Elmer (Hopkinton, MA), MDA-MB-231 cells were obtained from Duke Cell Culture Facility, a repository of ATCC cell lines that are available to Duke University researchers. The cell lines were authenticated by the DNA Analysis Sequencing facility at Duke University using Short Tandem Repeat (STR) DNA profiling. Cell lines used in the animal studies were verified to be murine pathogen free following IMPACT III murine pathogen analysis by IDEXX BioResearch (Columbia, MO). PC-3M-luc-C6 cells were cultured in Gibco MEM media modified with Earle's salts, L-glutamine (Waltham, MA) and supplemented with 10% heat inactivated-fetal bovine serum (FBS), 0.1 mM non-essential amino acids, 1 mM sodium pyruvate, and 1% penicillin/streptomycin. MDA-MB-231 cells were cultured in minimum essential medium Eagle (MEME) with Earle's salts, L glutamine, and sodium bicarbonate (Sigma Aldrich, St. Louis, MO) supplemented with 10% FBS, 1 mM sodium pyruvate, and 0.1 mM non-essential amino acids. 4.5 g.L-1 D-glucose (Sigma, St. Louis, MO), 10 mM HEPES (Invitrogen, Carlsbad, CA), 100 U.mL-1 penicillin/streptomycin (Gibco, Grand Island, NY) and 1 mM sodium pyruvate (Invitrogen, Carlsbad, CA). Cells were passaged every 2-3 days by enzymatic detachment with 0.05% trypsin/EDTA (Invitrogen, Carlsbad, CA).

***In vitro* Cytotoxicity.** Cell viability was quantified using the CellTiter-Glo luminescent cell viability kit (Promega, Madison, WI). 3000 cells were seeded in 80 µL of media in Greiner Bio-one white 96-well flat bottom cell culture plates (Cat no: 655098). The cells were cultured for 16-18 h before treatment with sub-nanomolar to high micromolar concentrations of free PTX, CP-PTX, and ABD-CP-PTX in 20 µL DPBS. After 72 h of drug treatment, 15 µL of CellTiter-Glo reagent was added to each well in the dark. Cells were incubated for 40 min at room temperature, and the luminescence was measured on a Victor3 microplate reader (Perkin Elmer; Waltham, MA). For each plate, cells treated with DPBS were defined as 100% viable. The percentage of viable cells in drug treated wells was calculated by normalizing the luminescence reading from each treatment well to the luminescence of DPBS treated cells. The 50% inhibitory concentration, IC_{50}, was determined by fitting the data to a four-parameter logistic fit in GraphPad Prism (GraphPad, San Diego, CA).

Tumor Regression. For tumor implantation, detached cells were washed twice in Minimum Essential Medium (MEM, 51200-038; Invitrogen; Carlsbad, CA) and resuspended in fresh MEM. For the orthotopic tumor model, tumor inoculation was performed by injecting 5 $x10^6$ MDA-MB-231 cells into the in mammary fat pad of mice. For the s.c. model 1 $x10^6$ PC3 cells were injected into the right flank. Tumors were allowed to grow until they reached a size of ~100 mm^3. Mice were randomly divided into different treatment groups. Each treatment group received a single dose of a drug via tail vein injection. Tumor growth and BW were monitored 2-3 times a week, and tumor dimensions were measured using hand-held digital slide calipers. Tumor volume was calculated using the equation: volume (mm^3) = $\pi/6*L*W^2$. Response to the treatments were evaluated by measuring the delay in tumor growth and survival. Mice were sacrificed when the tumor volume reached 1500 mm^3. All treated mice were given supportive care in the form of soft food (wetted powdered food) and HydroGel™(ClearH_2O, Portland, Maine) for 48 h post treatment. Mice were euthanized if the BW loss was greater than 15% of original BW for three consecutive measurements. All animals were treated in accordance with the National Institute of Health Guide for Supplemental Methods and Data for the Care and Use of Laboratory Animals under protocols approved by the Duke University Institutional Animal Care and Use Committee. Tumor regression study at Charles River was performed using the same protocol described above following their internal animal ethics guidelines. Studies at Duke were blinded (drug administration and observation). The study at Charles River was double-blinded (drug administration, observation, and analysis).

Synthesis and Characterization of Cyanine Labeled PTX-Conjugates. ABD-CP-PTX and CP-PTX were dissolved at 10 mg/mL in 1:1 sodium phosphate buffer (pH 7.4):DMF with 100 molar excess TCEP (100 mM, pH 7.4) for 0.5 h at room temperature. 20 molar excess of Cy5/Cy5.5-maleimide (Lumiprobe, Hunt Valley, MD) dissolved at 100 mg/mL in DMF was added to the polypeptide solution, and the conjugation reaction was allowed to proceed for 24 h at RT. Reaction mixture was diluted in PBS and excess dye was removed by sequentially passing the sample through Sephadex G-25 PD10 desalting column (3X, GE Healthcare, Pittsburgh, PA), followed by buffer exchange with 75% PBS and 25% acetonitrile by Amicon Ultra-15, 10 kDa MWCO, and finally, buffer exchanged into Milli-Q water and then lyophilized. The labeling efficiency was determined by resuspending it in PBS and to measuring Cy5/Cy5.5 concentration using UV-vis spectrophotometry (NanoDrop 1000, Thermo Fisher, Waltham, MA), using absorbance at 646 nm and ε =

250000 L cm^{-1} M^{-1} for the Cy5 fluorophore and absorbance at 684 nm and ε = 198000 L cm^{-1} M^{-1} for the Cy5.5 fluorophore.

Pharmacokinetics and Tumor Accumulation. For pharmacokinetic study, a single dose of Cy5 labeled ABD-CP-PTX and CP-PTX, at 5-50 mg/Kg B.W. PTX equivalent was administered i.v. to healthy athymic nude mice. Cy5 concentration was kept constant at 10 µM across all doses by doping with unlabeled conjugate. Over three days, 10 µL of blood was collected via tail vein at indicated time points into 40 µL of heparin (1000 U/mL) and centrifuged at 5000 x g for 5 min at 4 °C. The supernatant was collected and supplemented with 25% acetonitrile. 50 µL of the sample was then loaded into a clear bottom 384 well plate (Corning, NY) and read on a Typhoon 9410 Variable Mode Imager (GE Healthcare, Pittsburgh, PA) using the following settings: 450 PMT, 3 mm focal length. The amount of polypeptide remaining in circulation was calculated using a standard curve for Cy5. The blood concentration versus time data was analyzed with a standard two-compartment PK model using PK solver to determine PK parameters.[8] For in vivo tumor accumulation, mice bearing orthotopic MDA-MB-231 tumors (~100 mm^3) were put on an irradiated 5V75 alfalfa-free rodent diet (LabDiets, St. Louis, MO) to reduce autofluorescence associated with chlorophyll that is present in regular rodent food. Cy5.5 labeled PTX nanoparticles were injected via the tail vein (50 µM Cy5.5, at a dose of 25 mg/Kg BW of PTX equivalent). Live mice were imaged on an IVIS Lumina instrument (Perkin Elmer) using 660/710 nm excitation and emission filters to visualize the tumor accumulation at 3 h, 6 h, 1 d, 3 d, 7 d, 9 d, 11 d and 15 d. To quantify the amount of nanoparticle accumulation in the tumors over time, a region of interest (ROI) was drawn to cover the tumor area, and the fluorescence intensity in the ROI was calculated using Living Image Software (Perkin Elmer). This fluorescence intensity value was then normalized to the average tumor volume to account for any variability in tumor size.

Data Analysis: Data are presented as mean and standard error of the mean (SEM) unless otherwise mentioned. All statistical analyses were performed using GraphPad Prism v.9.0 software (GraphPad, San Diego, CA). $P < 0.05$ was considered as statistically significant.

References:

[1] J. Bhattacharyya, J. J. Bellucci, I. Weitzhandler, J. R. McDaniel, I. Spasojevic, X. Li, C.-C. Lin, J.-T. A. Chi, A. Chilkoti, *Nat. Commun.* **2015**, *6*, 7939.

[2] P. J. Kraulisa, P. Jonasson, P. Å. Nygren, M. Uhlén, L. Jendeberga, B. Nilsson, J. Kördel, *FEBS Lett.* **1996**, *378*, 190.

[3] A. Jonsson, J. Dogan, N. Herne, L. Abrahmsén, P. Å. Nygren, *Protein Eng. Des. Sel.* **2008**, *21*, 515.

[4] J. Nilvebrant, S. Hober, *Comput. Struct. Biotechnol. J.* **2013**, *6*, e201303009.

[5] P. Yousefpour, J. R. McDaniel, V. Prasad, L. Ahn, X. Li, R. Subrahmanyan, I. Weitzhandler, S. Suter, A. Chilkoti, *Nano Lett.* **2018**, *18*, 7784.

[6] T. Etrych, M. Sírová, L. Starovoytova, B. Ríhová, K. Ulbrich, *Mol. Pharm.* **2010**, *7*, 1015.

[7] C. L. Zhao, M. A. Winnik, G. Riess, M. D. Croucher, *Langmuir* **1990**, *6*, 514.

[8] Y. Zhang, M. Huo, J. Zhou, S. Xie, *Comput. Methods Programs Biomed.* **2010**, *99*, 306.
